# Integrated Phytochemical, Antioxidant, and Chemometric Evaluation of Clinically Relevant *Ocimum tenuiflorum* Extracts from Diverse Geo-Climatic Regions

**DOI:** 10.3390/molecules31213909

**Published:** 2026-10-29

**Authors:** Totan Das, Ishita Biswas, Satyajit Maity, Sayani Maji, K. Karunaidhasan, V. Anusuba, Digvijay Verma, Gurudev Choubey, Arti Soren, K. C. Muraleedharan, Subhash Kaushik, Shashikanth Jale, Bibaswan Biswas

**Affiliations:** 1 DAC Regional Research Institute, Kolkata 700035, West Bengal, India; totand946@gmail.com (T.D.); ishitabiswas159@gmail.com (I.B.); maitysatyajit1992@gmail.com (S.M.); sayanidonamaji@gmail.com (S.M.); gurudev.choubey@gmail.com (G.C.); arti_soren@rediffmail.com (A.S.); 2 Department of Pharmaceutical Technology, Jadavpur University, Kolkata 700032, West Bengal, India; 3 Centre of Medicinal Plants & Research in Homoeopathy, Ooty 643209, Tamil Nadu, India; karunaithasan1998@gmail.com (K.K.); anusubavr@gmail.com (V.A.); jaleshashikanth2000@gmail.com (S.J.); 4 Central Council for Research in Homoeopathy, New Delhi 110058, India; digvijayccrh@gmail.com (D.V.); drkcmurali@gmail.com (K.C.M.); subhashkaushik@ccrhindia.nic.in (S.K.)

**Keywords:** *Ocimum tenuiflorum*, antioxidant activity, rosmarinic acid, pharmacopeia, HPTLC, chemometrics

## Abstract

**Background/Objectives:** *Ocimum tenuiflorum* (OT) exhibits diverse therapeutic effects linked to its antioxidant activity. Studies integrating phytochemical variability, functional antioxidant localization, and chemometric evaluation of Pharmacopeia-Relevant Extracts (PREs) remain limited. This study investigated phytochemical variability and antioxidant-associated profiling among PREs of OT from geo-climatic regions and commercial sources. **Methods:** PREs were investigated through High-Performance Thin-Layer Chromatography (HPTLC) fingerprinting, HPTLC-2,2-diphenyl-1-picrylhydrazyl (DPPH) bioautography, HPTLC quantification, Total Phenolic and Flavonoid Content (TPC and TFC), DPPH-IC50 and Ferric Reducing Antioxidant Power (FRAP) assays, correlation statistics, and chemometric analyses. **Results:** Rosmarinic acid (RA) and ursolic acid (UA) were detected across all PREs; eugenol was not detected but detected in non-polar extracts. HPTLC-DPPH bioautography exhibited antioxidant-associated localization for RA but not UA. RA concentration, TPC, TFC, DPPH-IC50, and FRAP showed substantial variability among PREs. RA correlated more strongly than TPC with antioxidant endpoints: positively with FRAP and inversely with DPPH-IC50; TFC correlated non-significantly with the dataset. Geo-climatic variability was linked to differences in RA, TPC, IC50, and FRAP, with TFC not reaching significance, and varietal differences remained non-significant. Chemometric analyses demonstrated distinguishable clustering among PREs. **Conclusions:** This study highlights observational trends in geo-climatic phytochemical variation, identifying RA as a major antioxidant-associated marker under the present conditions.

## 1. Introduction

Even today, medicinal plants are one of the most important sources of therapeutic materials [1]. In spite of the historical and current significance of natural products toward human health, contemporary healthcare systems have increasingly shifted from plant-based therapeutics toward isolated natural products and synthetic pharmaceuticals [2]. This shift is primarily attributed to the rapid action, simpler standardization, precise dosing, and reproducible clinical validation of isolated compounds and synthetic pharmaceuticals [3].

Unlike synthetic or isolated compounds, the final chemical compositions of plant-based formulations are heavily dependent on their growing conditions, i.e., agro-climatic conditions, soil characteristics, agricultural stress factors, plant age, and seasonal variations [4,5,6]. Furthermore, it is well-established that post-harvest handling and storage profoundly affect the secondary metabolite profiling of the formulations [7]. As a result, the same preparation or formulation prepared from the same plant species sometimes differs considerably [8]. Added to this, herbs in industrial setups often come from diverse geographical regions with significantly varying growing conditions and distinct post-harvest handling, alongside a sometimes uncontrollable supply chain, making the situation more heterogeneous. This complicates the validation of the clinical efficacy.

The sourcing, standardization, quality control, and quality assurance of herb-based medicinal preparations are intrinsically challenging due to their extremely complex nature, making full characterization of herbal formulations elusive even today [9,10]. In order to address this issue, herbal drug standardization often relies on the quantification of different bioactive compounds present in those herbs and quantification of prominent secondary metabolites, i.e., phenols, flavonoids, and saponins [11,12]. The resulting chemical heterogeneity of such work is often addressed by comparing several samples simultaneously to gauge the possible deviation [12,13]. There were instances where researchers even compared domestically produced materials with foreign-origin materials [8,14]. Furthermore, advanced chemometric and machine learning approaches, such as principal component analysis, linear discriminant analysis, support vector machines, and random forest classification, are applied to process complex multivariate data, enabling statistical differentiation, feature extraction, and robust chemical characterization of heterogeneous samples [15]. Even though considerable efforts have been made to address this diversity and heterogeneity, studies dealing with applied settings are often limited, if not rare, particularly those dealing with extracts relevant or close to pharmacopeial preparation. Many of the existing studies deal with a single sample from each location or multiple samples collected from a single site or nearby geographical areas. Additionally, these investigations chiefly follow in-house controlled sourcing and strictly standardized post-harvest handling. Moreover, such studies usually use laboratory-defined plant extracts rather than pharmacopeia-aligned extracts originating from a plant source [16,17].

Beyond technical hurdles, the herbal drug industry faces socio-economic and logistical challenges stemming from decentralized sourcing networks, making uniform cultivation, standardized post-harvest handling, and fully traceable supply chains difficult to maintain under real-world conditions [18]. These unregulated variations in farming and processing practices introduce unmeasured agricultural stress and phytochemical inconsistency.

Therefore, it was envisioned that comparative quantitative evaluation of phytochemical variability among pharmacopeially relevant preparations originating from different geographical regions, consisting of taxonomically authenticated samples together with commercially sourced samples, may lay the foundation of a practical strategy for advancement toward robust herbal drug standardization.

Conventionally, herbal drug quality control deals with different ash values, extractive values, pH of the preparation, Ultraviolet–Visible (UV–VIS) spectra, Total Phenolic Content (TPC), Total Flavonoid Content (TFC), Thin-Layer Chromatography (TLC), High-Performance Thin-Layer Chromatography (HPTLC) fingerprinting, etc. [13]. Although these provide preliminary information, they fail to capture the important implications of these physicochemical and phytochemical results. On the other hand, broad-spectrum untargeted metabolite analysis methods, namely Gas Chromatography–Tandem Mass Spectrometry (GC–MS/MS) and Liquid Chromatography–Tandem Mass Spectrometry (LC–MS/MS), although they are exploratory in nature, may be used as the preliminary target identification and usually provide a tentative and putative chemical profiling. Hence, such tentative and putative chemical profiling results should be verified through orthogonal structural confirmation or characterization of isolated purified compounds, not only to improve their translational relevance in pharmaceutical applications but also to ensure greater analytical accuracy in metabolite identification [19,20]. Therefore, due to the inherent heterogenous nature of the herbal samples, it is important to couple in-depth analytical studies with chemometric modeling for a more comprehensive phytochemical profiling beyond its qualitative observations.

*Ocimum tenuiflorum* L. (OT) (previously known as *Ocimum sanctum* L.) or Holy basil is one of the most important medicinal plants in Southeast Asia, especially in India. Its immense medicinal properties have deep cultural significance, especially in India, where it is spiritually revered and hailed as “Elixir of Life” [21]. Its significance extends beyond traditional and ethnomedicinal uses, with modern phytopharmaceutical research recognizing its diverse biological benefits [22,23,24]. Research has revealed the plant’s diverse pharmacological potential, including significant antimicrobial, anti-inflammatory, anticancer, antidiabetic, and immunomodulatory activities [25,26,27]. OT also shows prominent cardioprotective, hepatoprotective, neuroprotective, and renoprotective activities [28,29,30,31]. Interestingly, the central mechanism of action of OT appears to stem from its antioxidant activity, making it a unifying pathway underlying its versatile bioactivities. Recent studies on OT suggest that its anti-inflammatory, anticancer, hepatoprotective, cardioprotective, and neuroprotective effects are linked with oxidative stress, including Reactive Oxygen Species (ROS) generation/scavenging, lipid peroxidation, and endogenous antioxidant defense mechanisms [26,29,32,33,34].

Considering its medicinal significance, several studies have been undertaken on this plant, including attempts to correlate various chemical parameters with its antioxidant effect. Agarwal et al. found antioxidant properties of ethyl acetate extracts of both OT and *Ocimum kilimandscharicum*. They also tentatively identified Rosmarinic Acid (RA), caffeic acid, and other caffeic acid derivatives through High-Performance Liquid Chromatography/Liquid Chromatography–Mass Spectrometry (HPLC/LC–MS) [35]. However, the study involved only one sample from each species, making the investigation fundamentally observational. Tomar et al. similarly associated the antioxidant potential of essential oils of OT and *Ocimum gratissimum* L. However, similar to Agarwal’s study, they used only one sample for each species [36]. Saelao et al. comprehensively correlated antioxidant properties with major classes of secondary metabolites, including phenolics, anthocyanins, and flavonoids, through chemometric analyses [37]. However, the study remained restricted to class-level phytochemical assessment without direct functional detection of antioxidant-active constituents. Chaudhary et al. also principally found results similar to those of Saelao et al., wherein extract fractions with high flavonoid and phenolic contents showed higher antioxidant properties [38]. Furthermore, recent developments in integrated metabolomic and chemometric investigations have unraveled phytochemical variability and multivariate differentiation in *Ocimum* species [39]. However, many currently reported studies still lack comprehensive isolation, orthogonal structural confirmation, and direct functional evaluation of specific phytochemical markers, particularly in relation to antioxidant activity under pharmacopeia-relevant extraction conditions.

To address the existing research gaps, this present study profiled and identified chemical markers in an OT tincture prepared in accordance with pharmacopeial standards, sourced from different geographical regions alongside commercially sourced plant samples [40]. This specific pharmacopeia was selected because its adherence to single-plant-based tincture preparations provides a scientifically coherent framework for targeted analytical validation, avoiding the complexities of multi-herb formulations. Comparative HPTLC fingerprinting identified RA and Ursolic Acid (UA) as ubiquitous markers, with RA showing a strong radical scavenging profile in the HPTLC-2,2-diphenyl-1-picrylhydrazyl (DPPH) assay, which was further confirmed through orthogonal structural characterization. RA was quantified using a validated HPTLC method, and antioxidant capacity was evaluated through DPPH and Ferric Reducing Antioxidant Power (FRAP) assays. Phytochemical variability was monitored via Total Phenolic Content (TPC) and Total Flavonoid Content (TFC). These phytochemical and antioxidant parameters were statistically correlated and further examined for geographical and varietal influences. The resulting patterns were evaluated through chemometric analyses to assess sample clustering across the studied geo-climatic regions.

## 2. Results

### 2.1. Phytochemical Standard Marker Screening and Detection of Antioxidant-Associated Markers

**Figure 1:**
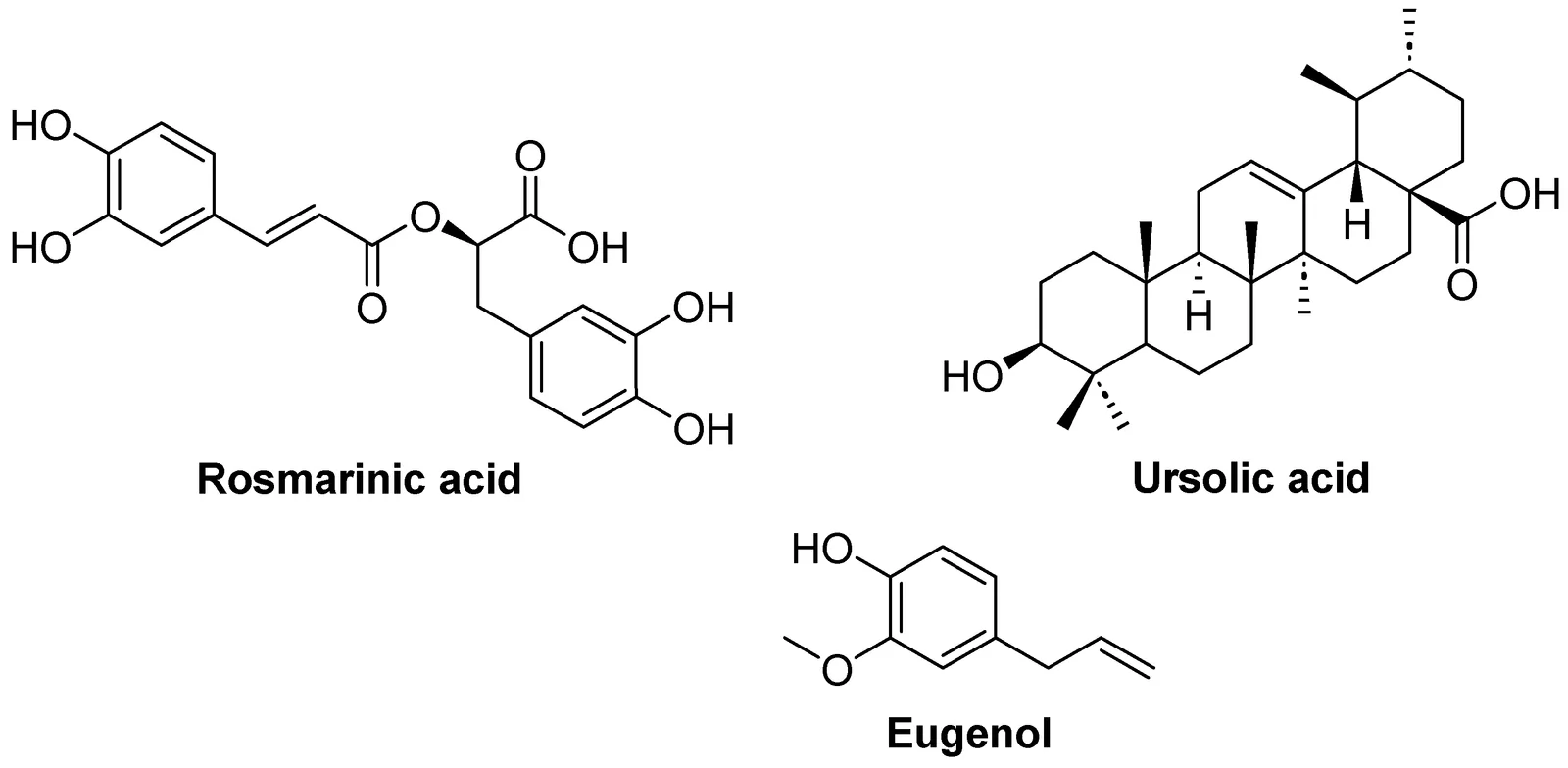
Structures of Rosmarinic Acid (RA), Ursolic Acid (UA), and eugenol.

**Figure 2:**
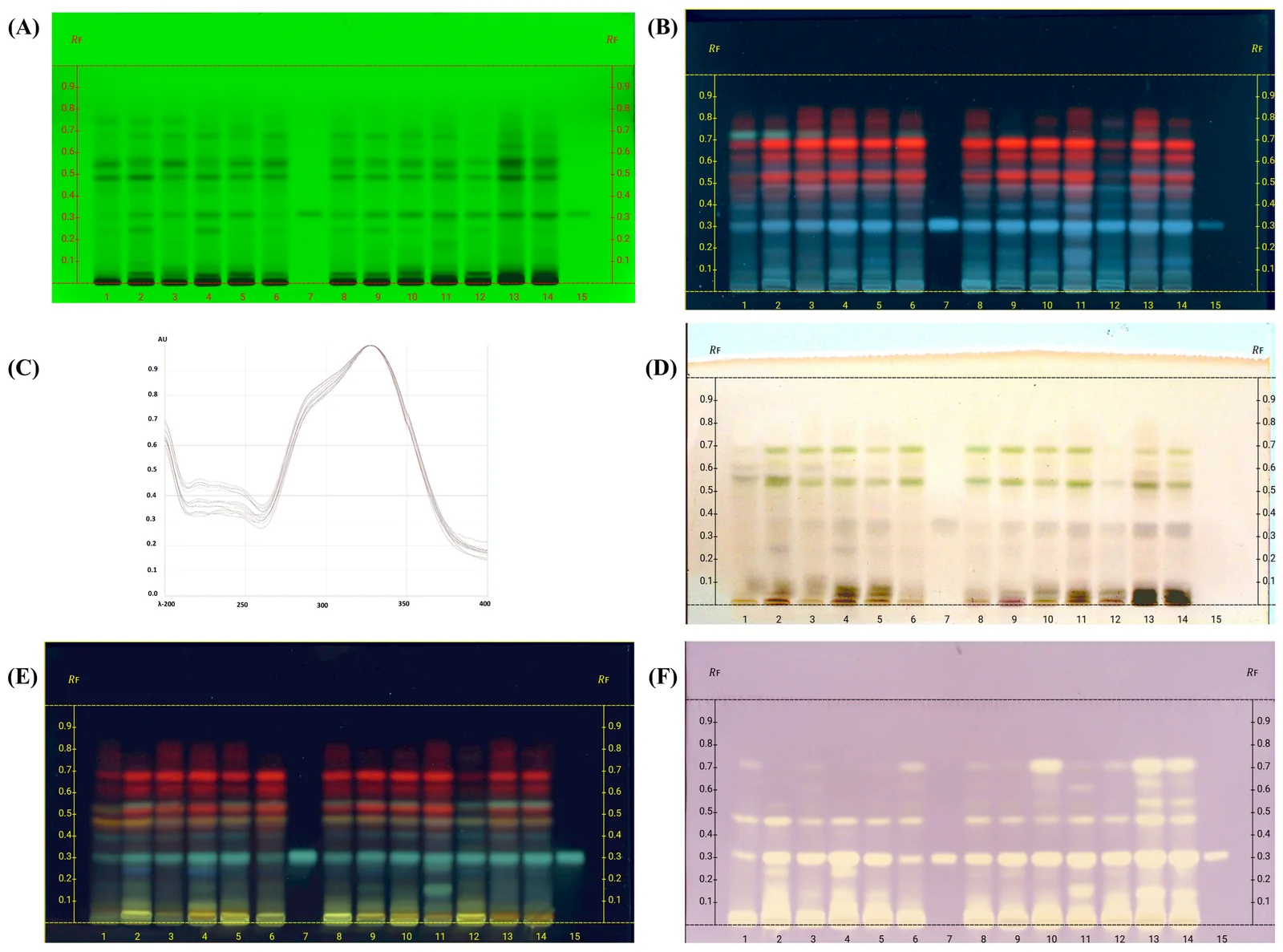
HPTLC fingerprinting and antioxidant profiling of PREs. (**A**) 254 nm chromatogram. (**B**) 366 nm chromatogram. (**C**) Overlaid UV spectra of RA bands from PREs and RA reference standard. (**D**) Ferric chloride derivatization. (**E**) NP/PEG derivatization. (**F**) HPTLC-DPPH bioautography. Tracks 1–6: PREs of 10612, 10835, 10844, 10876, 10909, and 11009; Tracks 8–14: PREs of 11018, 11114, 11118, MR1, MR2, MR3, and MR4; Tracks 7 and 15: RA reference standard. **Abbreviations:** HPTLC, high-performance thin-layer chromatography; PREs, pharmacopeia-relevant extracts; RA, rosmarinic acid; NP, natural product reagent; PEG, polyethylene glycol; DPPH, 2,2-diphenyl-1-picrylhydrazyl.

**Figure 3:**
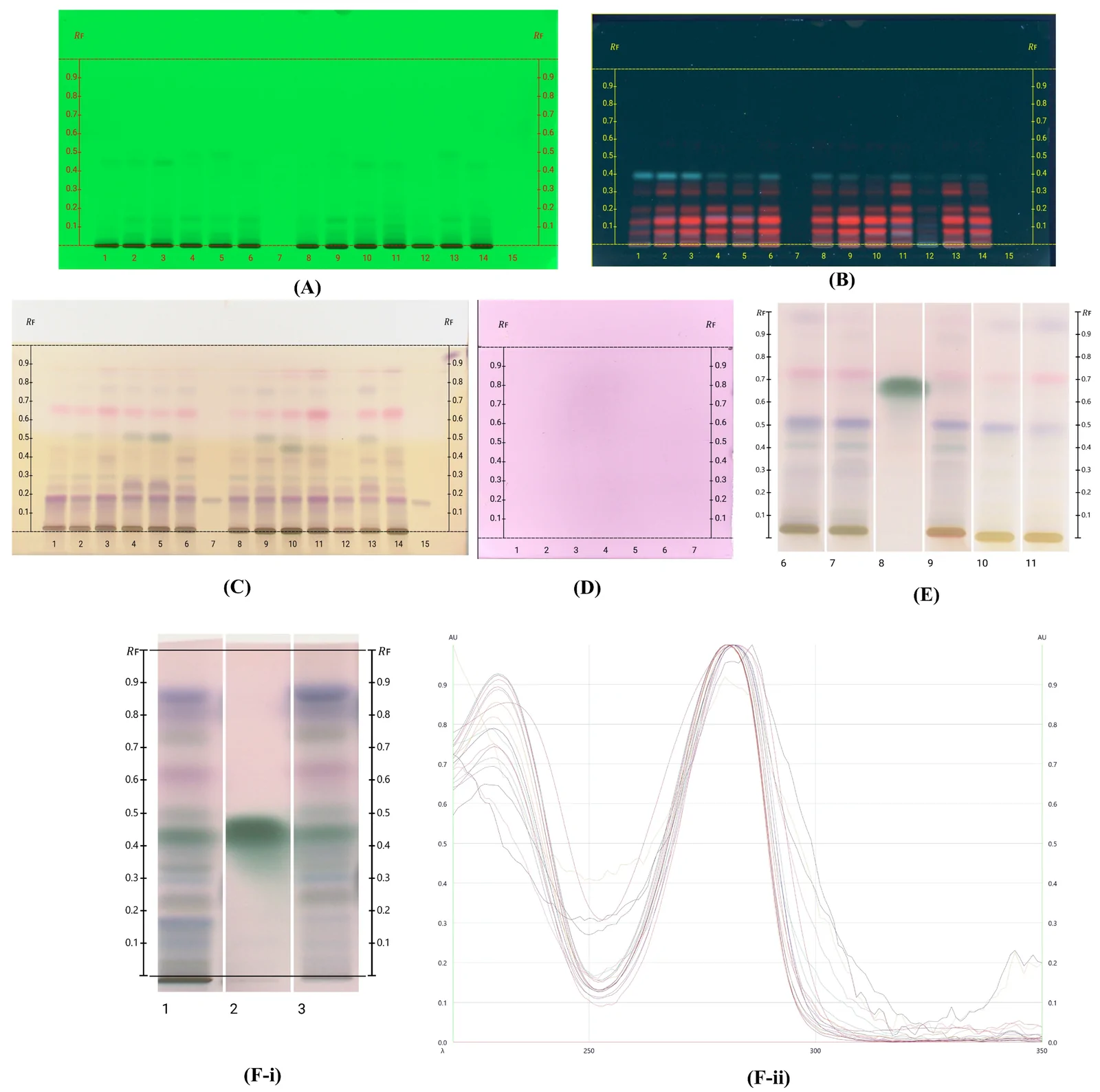
HPTLC fingerprinting and marker analysis of PREs. (**A**) Chromatogram under 254 nm. (**B**) Chromatogram under 366 nm. (**C**) ASR derivatization. Tracks 1–6: PREs of 10612, 10835, 10844, 10876, 10909, and 11009; Tracks 8–14: PREs of 11018, 11114, 11118, MR1, MR2, MR3, and MR4; Tracks 7 and 15: UA reference standard. (**D**) HPTLC-DPPH bioautography of UA reference standard. (**E**) Eugenol detection: Tracks 6–7: PREs of 10844 and 10876; Track 8, eugenol reference standard; Tracks 9–11: PREs of 11009, 11114, and 11118. (**F**) (**i**) Eugenol detection in 10876: Track 1, DCM extract of 10876; Track 2, eugenol reference standard; Track 3, pentane extract of 10876. (**ii**) Overlaid UV spectra of eugenol bands from DCM and pentane extracts of 10876 and eugenol reference standard. **Abbreviations:** HPTLC, high-performance thin-layer chromatography; PREs, pharmacopeia-relevant extracts; UA, ursolic acid; ASR, anisaldehyde sulphuric acid reagent; DPPH, 2,2-diphenyl-1-picrylhydrazyl; DCM, dichloromethane.

Thirteen Pharmacopeia-Relevant Extracts (PREs) were investigated in this study: nine field-collected samples, identified hereafter by their voucher Field numbers, and four commercially sourced samples, designated MR1, MR2, MR3, and MR4. Full collection details, geo-climatic zone assignments, and voucher information are provided in Section 4.1 and Table 11. Possible phytochemical markers—Rosmarinic Acid (RA), Ursolic Acid (UA), and eugenol (Figure 1)—present in the plant extracts prepared from all 13 samples were screened using HPTLC fingerprinting. Several derivatizing agents, e.g., Anisaldehyde Sulphuric acid Reagent (ASR), Natural Products-Polyethylene Glycol reagent (NP/PEG), and ferric chloride (Figure 2 and Figure 3), were employed for the detection of key secondary metabolites, along with densitogram and spectral analyses.

The solvent system comprising toluene, ethyl acetate, and formic acid in a ratio of 5:4:1 (*v*/*v*/*v*) afforded well-resolved bands in the polar region, and RA was detected through R_f_ and spectral matching (Figure 2A–F). Hexane and ethyl acetate in a ratio of 4:1 (*v*/*v*) provided satisfactory separation in the non-polar region, enabling the detection of UA (Figure 3A–C). HPTLC screening of all thirteen OT PREs demonstrated the consistent presence of RA and UA across the investigated samples (Figure 2A,B,D–F and Figure 3C).

However, eugenol, a known biologically active phytochemical of OT, could not be detected in the PREs through HPTLC, although the corresponding pentane and dichloromethane (DCM) extracts of raw plant material of 10876, prepared analogously, showed its substantial presence, as evident by its R_f_ and spectral matching (Figure 3E,F(i,ii)).

These observations suggest extraction-selective phytochemical partitioning within the pharmacopeia-relevant extract system. This finding is consistent with a recent GC–MS study in which eugenol was a major constituent (32–35%) of OT essential oil [41]. Its recovery mainly in non-polar extracts in the present study is consistent with its lipophilic nature.

Additionally, HPTLC chromatograms (Figure 2A,B,D–F and Figure 3A–C,E,F(i)) show several bands besides RA, UA, and eugenol. Such additional bands are expected given the known, chemically rich and complex composition of OT. Reporting chromatograms with multiple bands without full characterization of each is a standard, previously documented practice for this species/genus [12]. The present study deliberately adopted a targeted marker approach, focusing on RA, UA, and eugenol because of their relevance to standardization and pharmacology and because they are commercially available as certified reference standards, permitting HPTLC-based R_f_ and spectral matching.

Marker screening was based on R_f_ values and UV spectra compared with certified reference standards, along with derivatization using specific reagents (ASR, NP/PEG, and ferric chloride). This allowed the initial detection of RA, UA, and eugenol. Since eugenol was not detected in the PREs, it was not studied further. RA and UA were detected in all 13 PREs. However, UA did not show antioxidant activity, which was the main focus of this study, whereas RA produced a clear bleached zone in HPTLC-DPPH bioautography. The identity of RA was therefore further confirmed by its isolation from the PREs and characterization using ^1^H-NMR, ^13^C-NMR, and HRMS.

Since both RA and UA were consistently detected in all 13 PREs, an HPTLC-DPPH assay was further performed to locate the antioxidant-active constituents. RA showed strong DPPH-radical scavenging activity, while UA did not show any detectable antioxidant activity in the bioautography assay, even when the same assay was carried out with pure UA (Figure 2F and Figure 3D). Additional bleached active zones were also observed for PREs in addition to RA, indicating that additional antioxidant constituents alongside RA contribute to the overall radical scavenging profile.

### 2.2. Molecular Docking Study

Although molecular docking alone does not directly establish biological activity or antioxidant efficacy under physiological conditions, the relative antioxidant and possible bioactive potentials of RA and UA were additionally explored through preliminary molecular docking analysis. The in silico observations (Table 1) demonstrated comparatively higher binding affinities for RA compared to UA. It must be emphasized that these computational scores do not confirm in vitro or in vivo efficacy for the listed therapeutic activities but rather serve as supplementary theoretical support alongside the HPTLC-DPPH observations for choosing the more important marker between RA and UA. Corresponding reference standard complexes with target proteins listed in Table 1 are given in Figures S1 and S2. Together with the HPTLC-DPPH observations, these findings led to the selection of RA as the principal marker compound for subsequent quantitative and chemometric investigations.

### 2.3. Quantification and Characterization of Rosmarinic Acid (RA)

**Figure 4:**
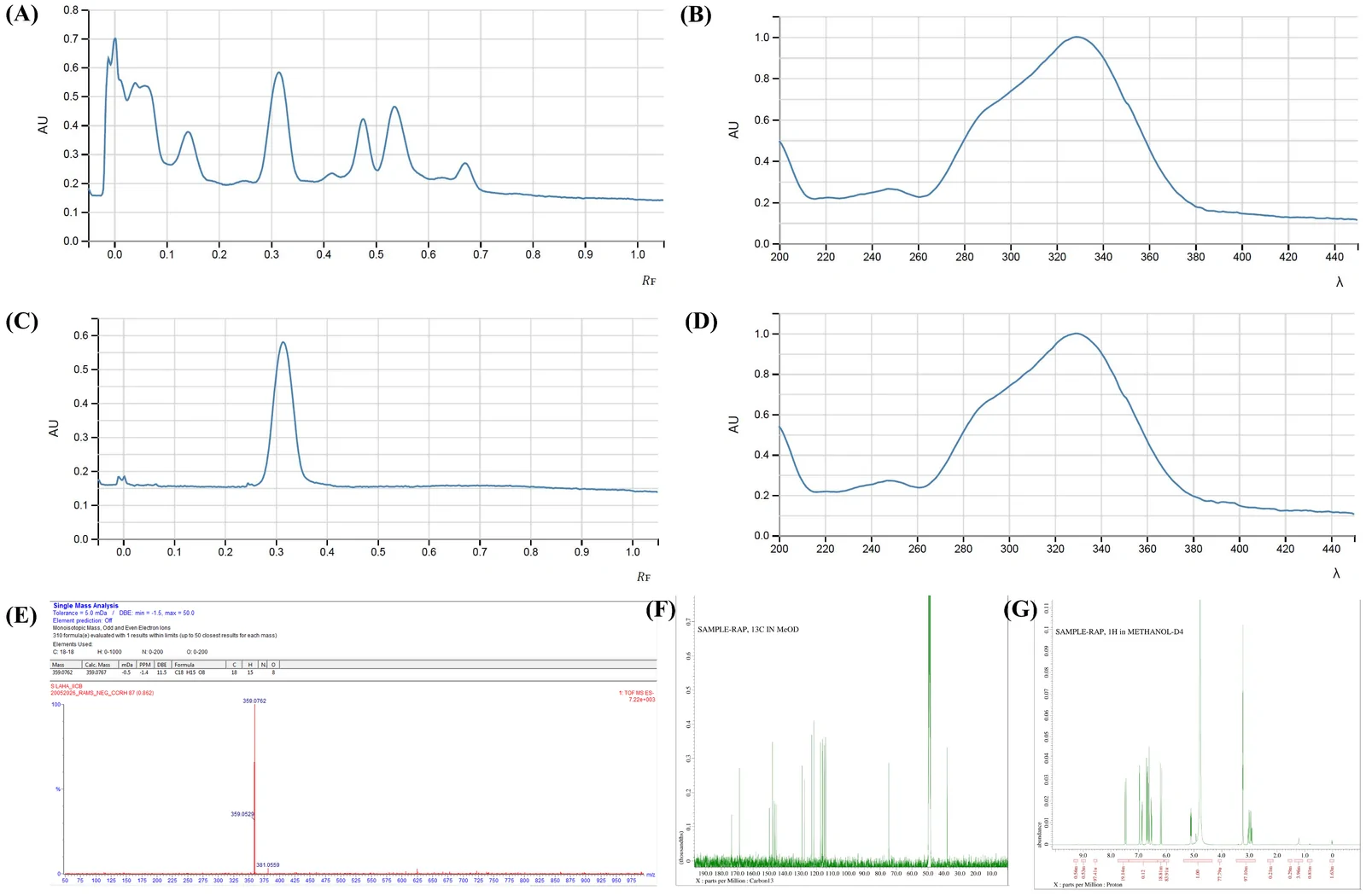
HPTLC densitogram and structural characterization of RA. (**A**) Densitogram of PRE of 10876 at 325 nm. (**B**) Absorption spectrum of the PRE of 10876 spot corresponding to RA. (**C**) Densitogram of RA reference standard. (**D**) Absorption spectrum of RA reference standard. (**E**) HRMS (−ESI) spectrum of isolated RA. (**F**) ^13^C-NMR spectrum of isolated RA in d_4_;-methanol. (**G**) ^1^H-NMR spectrum of isolated RA in d_4_;-methanol. **Abbreviations:** HPTLC, high-performance thin-layer chromatography; PRE, pharmacopeia-relevant extract; RA, rosmarinic acid; HRMS, high-resolution mass spectrometry; ESI, electrospray ionization; NMR, nuclear magnetic resonance.

Following the marker evaluation, RA quantification was done through HPTLC densitometry at R_f_ ≈ 0.30 at 325 nm (Figure 4A–D). Also, a sample calibration curve of RA by HPTLC densitometry at 325 nm is shown in Figure S3.

The outcome of the quantification of RA (in μg/mL) is summarized in Table 2. The RA concentration ranged from 14.54 ± 0.07 µg/mL (Table 2, Entry 6: 11009) to 169.40 ± 0.58 µg/mL (Table 2, Entry 13: MR4). Other Indus Plain/Semi-Arid region samples also showed high RA concentrations: 97.81 ± 0.30 µg/mL and 149.00 ± 2.10 µg/mL respectively for 11114 and 11118 (Table 2, Entry 8 and 9). The lowest RA concentration was found in sample 11009, a Western Ghats sample (Table 2, Entry 6). Similar lower concentrations were also observed in the other two W samples, 10612 and 11018, having concentrations of RA 28.82 ± 0.29 µg/mL and 35.07 ± 0.23 µg/mL (Table 2, Entries 1 and 7).

The quantification method was validated comprising sensitivity through Limit Of Detection (LOD), Limit Of Quantification (LOQ), linearity, intraday precision, interday precision, recovery, and robustness. For RA, linearity was found to be between 250 and 600 ng, having R^2^ > 0.99 (Table 3, No. 1, 2, and 3). The LOD and LOQ values were found to be 38.85 ng and 117.73 ng, respectively, via regression (Table 3, No. 5 and 6). The RA was further orthogonally confirmed by HRMS (**−ESI**), ^13^C-NMR, and ^1^H-NMR analyses through comparison with previously reported literature data (Figure 4E–G) [42]. The full spectra are given in Figures S4, S5 and S6 respectively.

The intraday (*n* = 3) and interday (*n* = 3) analysis was done with the PRE itself instead of using standards due to the significant chemical complexity of the matrix. The recovery was found to be within ±2%. When the saturation time was changed by ±20% and the development length was changed by ±12.5%, the R_f_ was not appreciably changed. However, when the solvent system was changed to toluene, ethyl acetate, and formic acid in ratios of 5.2:3.8:1 (*v*/*v*/*v*) and 4.8:4.2:1 (*v*/*v*/*v*), the R_f_ value remained 0.29, while in the latter case, it became 0.33, respectively, without affecting the purity of the spot, indicating the robustness of the method. The intra- and interday (*n* = 3) for the %RSD/%CV were found to be around 0.5 for both cases.

### 2.4. Total Phenolic and Total Flavonoid Content

The Total Phenolic Content (TPC) and Total Flavonoid Content (TFC) of all the extracts were determined by Folin-Ciocalteu and aluminum chloride colorimetric assays, respectively. In both cases, calibration curves were constructed by plotting absorbance against the final concentration of the standards in the reaction mixture. The 10-times diluted PREs produced absorbance values within the linear range of the corresponding calibration curves. Both respective standard calibration curves demonstrated strong linearity, with correlation coefficients exceeding 0.99 (TPC: 0.995; TFC: 0.999). The measured TPC and TFC values for PREs are given in Table 4. The calibration curves, R^2^ values for linear regression, and the linear regression equations for TPC and TFC measurements are given in Figures S7 and S8 respectively.

The TPC values ranged from 0.64 ± 0.048 mg GAE/mL in 11009 (Table 4, Entry 6) to 2.45 ± 0.008 mg GAE/mL in MR4 (Table 4, Entry 13), while the TFC values ranged from 0.63 ± 0.009 mg QE/mL for 10612 (Table 4, Entry 1) to 3.60 ± 0.020 mg QE/mL for MR3 (Table 4, Entry 12). Three Indus Plain/Semi-Arid region samples (11114, 11118, and MR4), along with 10876, showed synchronously high RA concentrations and generally higher TPC (Table 2 and Table 4, Entry 4,8,9, and 13). However, in contrast, no such observation could be made for TFC. For example, MR3 exhibited the highest TFC despite having only a moderate RA concentration (Table 2 and Table 4, Entry 12).

### 2.5. DPPH Radical Scavenging Activity

**Figure 5:**
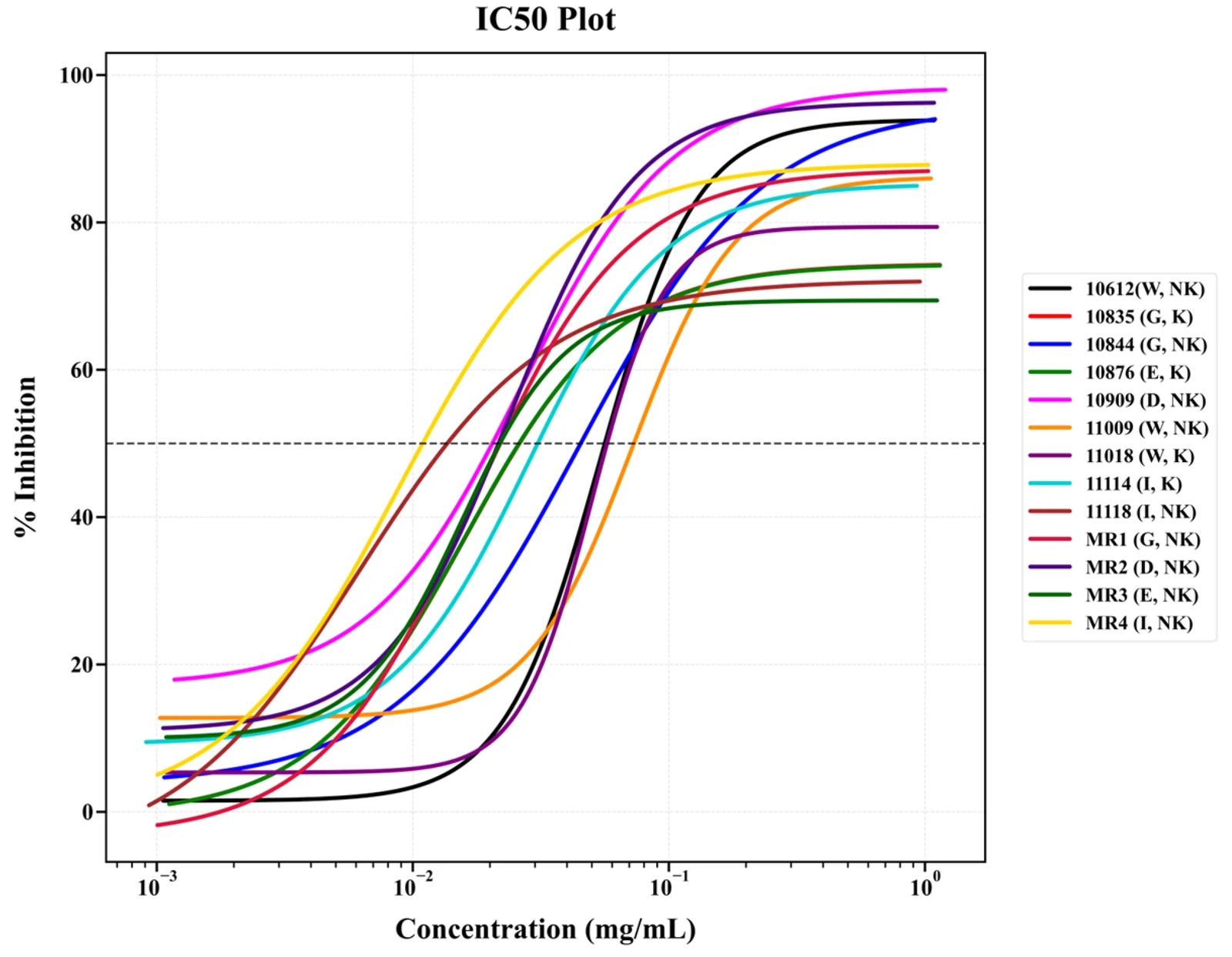
Fitted 4PL IC_50_ curves of PREs for DPPH inhibition. **Abbreviations:** 4PL, fitted four-parameter logistic; IC_50_, half-maximal inhibitory concentration; PREs, pharmacopeia-relevant extracts; DPPH, 2,2-diphenyl-1-picrylhydrazyl; W, Western Ghats; E, Eastern Ghats; D, Deccan; G, Gangetic Plain; I, Indus Plain/Semi-Arid; K, Krishna; NK, Non-Krishna.

Antioxidant profiling of the samples was performed by measuring the IC_50_ values of the DPPH assay, as HPTLC-DPPH bioautography demonstrated antioxidant effects in all PREs. A robust and comprehensive DPPH scavenging-based antioxidant profiling was obtained by measuring the % inhibition of the same volume of serially diluted PREs (10, 20, 40, 80, 160, 320, 640, 1280, 2560, 5120, and 10,240 times diluted), rather than using a single-point assay. Single-point assays often fail to capture the overall behavior of complex matrices, including possible single-phasic and biphasic antioxidant scavenging behavior that might be operative in complex plant extracts containing different kinds of antioxidant compounds. This assumption was further supported by the observation that the same volume of undiluted PREs produced almost complete inhibition in several cases. The results of IC_50_ values are given in Table 5, and the fitted 4PL dose–response curves (generated by Python 3.14) for all 13 PREs are given in Figure 5.

The IC_50_ values for the samples under study were found to be between 0.0061 ± 0.0015 and 0.0719 ± 0.0013 mg/mL, i.e., more than 10-fold variability was observed (Table 5, Entries 9 and 6). Among all samples, the strongest DPPH radical scavenging activity was shown by 11118, followed by MR4 (Table 5, Entries 9 and 13). The lowest DPPH activity was demonstrated by 11009 (Table 5, Entry 6). The IC_50_ of ascorbic acid was found to be 0.0112 mg/mL. Importantly, 11118 and MR4 exhibited lower IC_50_ values by mass than ascorbic acid. This enhanced activity may stem from the additive contribution of multiple antioxidant constituents identified alongside RA in the HPTLC-DPPH bioautography (Section 2.1).

### 2.6. Ferric Reducing Antioxidant Power (FRAP)

The obtained FRAP values ranged from 0.48 ± 0.009 (Table 6, Entry 1) to 1.97 ± 0.033 (Table 6, Entry 9) mg of Trolox Equivalent (TE)/mL. The calibration curve of Trolox, together with the corresponding linear regression equation and R^2^ values, is presented in Figure S9. Comparable to the DPPH assay, substantial variability was observed among the PREs. However, unlike the DPPH assay, the difference between the highest and lowest FRAP values was approximately four-fold. Such comparison, however, should be interpreted cautiously, considering the differences in the experimental design and mechanisms of the redox processes involved. Overall, the DPPH inhibition experiment was substantiated by the FRAP assay showing consistent antioxidant behavior among the PREs. The Indus Plain/Semi-Arid region showed high FRAP values ranging from 1.51 ± 0.035 to 1.97 ± 0.033 mg of TE/mL (Table 6, Entries 8, 9, and 13). Conversely, values for the Western Ghats samples were consistently lower, ranging from 0.48 ± 0.009 to 0.83 ± 0.003 mg of TE/mL (Table 6, Entries 1, 6, and 7).

### 2.7. Integrated Phytochemical and Antioxidant Profiling of PREs

The comparative profiling of PREs was integrated by ranking the average RA concentrations, TPC, TFC, IC_50_, and FRAP values in Table 7. Values in parentheses specify the ranking of the particular PRE for the corresponding parameter; for IC_50_, rank 1 represents the lowest value, whereas for other parameters, higher values correspond to higher ranks. Importantly, it should be noted that the rankings in Table 7 show the order of the mean values. However, adjacent ranks do not necessarily represent statistically significant differences between samples, as indicated by the one-way ANOVA and Tukey’s HSD post hoc test (superscript letters, Table 2, Table 4, Table 5 and Table 6). Among all PREs, sample 11118 (Table 7, Entry 9) showed the highest overall antioxidant profile, with the highest FRAP activity (ranking 1) and lowest IC_50_ value (ranking 1). The ranking of the same 11118 for RA concentration is 2, and for TPC and TFC, it is 3. Similarly, the commercial sample from the same geographic region I, MR4 (Table 7, Entry 13), exhibited the highest RA concentration and TPC. The sample ranked 2nd for the FRAP activity and IC_50_. However, despite these strong antioxidant-associated parameters, MR4 simultaneously demonstrated one of the lowest TFC values (ranking 12). Conversely, sample 11009 (Table 7, Entry 6) ranked lowest in RA concentration, TPC, and IC_50_ (ranking 13) while ranking 2nd lowest in FRAP (ranking 12). However, its TFC ranking was moderate: 7th among 13 studied PREs. One of the most interesting rankings is that of 10876 (Table 7, Entry 4). Its rankings in RA concentration, TPC, TFC, IC_50_, and FRAP were 4, 2, 2, 4, and 5, respectively, showing consistent ranking overall. Tukey’s HSD test showed that some adjacent ranks were not significantly different. This was particularly clear for IC_50_, where four samples formed a statistically indistinguishable group. Therefore, the rankings in Table 7 should be viewed as general trends rather than exact pairwise differences. Qualitatively, it seemed from the rankings that RA concentration, TPC, IC_50_, and FRAP were somewhat correlated, and such correlation for TFC might be weaker or absent, and data also suggest geographical influence on these parameters. However, the rankings were not monotonic among those parameters, necessitating statistical analyses to test their significance.

### 2.8. Correlation Analysis Between Phytochemical Parameters and Antioxidant Activity

Shapiro–Wilk normality analysis (Table 8) indicated that, among the measured parameters, only RA concentration significantly deviated from normal distribution (*W* = 0.855, *p* = 0.033) (Table 8, Entry 1), while the others, TPC, TFC, IC_50_, and FRAP, showed *p*-values > 0.05, suggesting approximate normal distribution characteristics under the present dataset (Table 8, Entry 2–5).

As there are observational trends among phytochemical parameters and antioxidant activity, both Pearson correlation and Spearman analyses were performed (Table 9).

**Figure 6:**
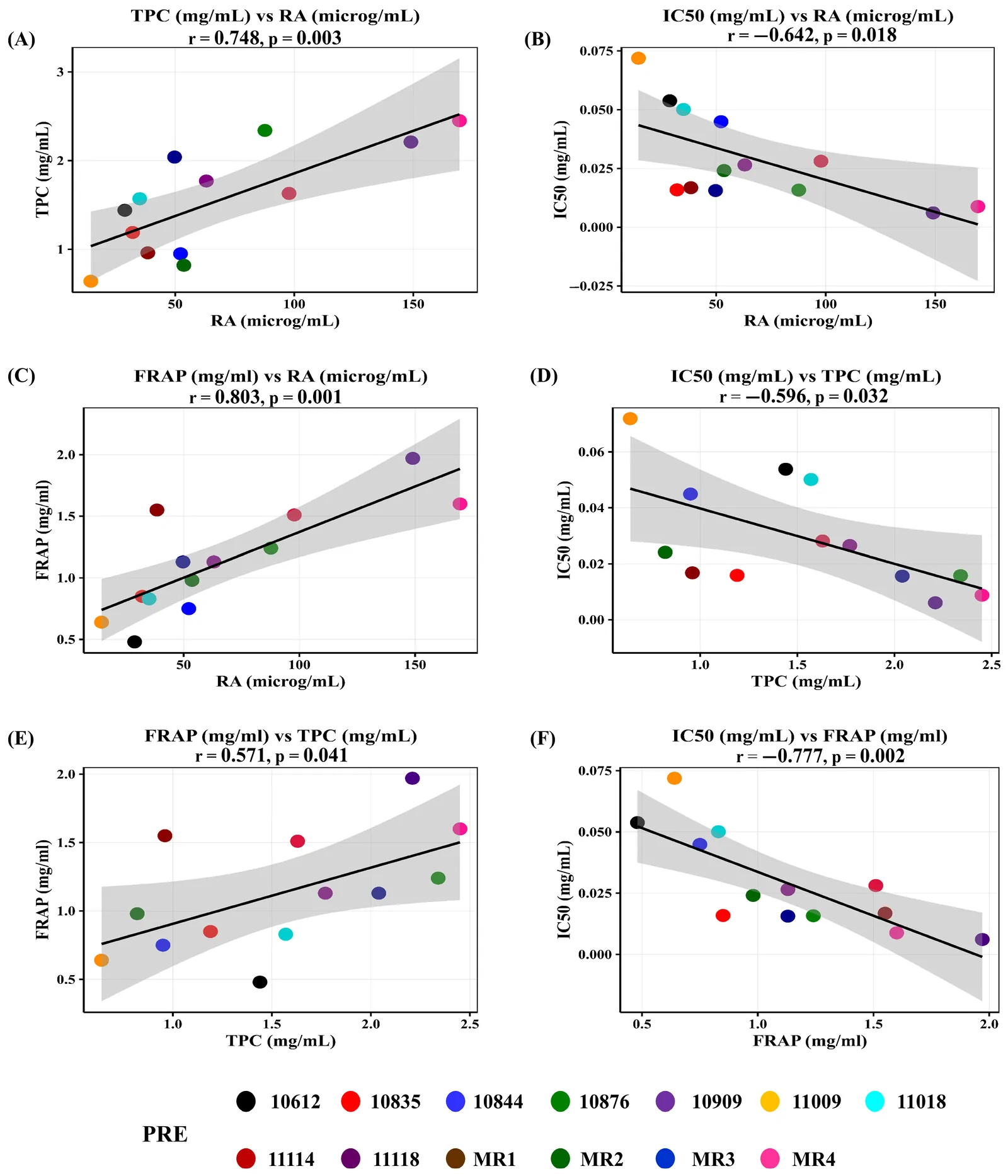
Graphical representation of statistically significant correlations. (**A**) TPC (mg GAE/mL) vs. RA (µg/mL). (**B**) IC_50_ (mg/mL) vs. RA. (**C**) FRAP (mg TE/mL) vs. RA (µg/mL). (**D**) IC_50_ (mg/mL) vs. TPC (mg GAE/mL). (**E**) FRAP (mg TE/mL) vs. TPC (mg GAE/mL). (**F**) FRAP (mg TE/mL) vs. IC_50_ (mg/mL). **Abbreviations:** TPC, total phenolic content; GAE, gallic acid equivalent; RA, rosmarinic acid; IC_50_, half-maximal inhibitory concentration; FRAP, ferric reducing antioxidant power; TE, Trolox equivalent.

The statistically significant correlations among phytochemical parameters and antioxidant activity are graphically represented in Figure 6.

RA concentration and TPC showed a positive correlation as demonstrated by Pearson (r = 0.748, *p* = 0.003) and Spearman (*ρ* = 0.692, *p* = 0.009) analyses (Table 9, Entry 1, Figure 6A). Likewise, RA concentration showed a significant positive correlation with FRAP through Pearson (r = 0.803, *p* = 0.001) and Spearman (*ρ* = 0.806, *p* = 0.001) analyses (Table 9, Entry 4, Figure 6C). On the contrary, RA concentration showed a significant negative correlation with IC_50_ as exhibited through both Pearson (r = −0.642, *p* = 0.018) and Spearman (*ρ* = −0.648, *p* = 0.017) analyses (Table 9, Entry 3, Figure 6B). TPC further demonstrated a significant negative correlation with IC_50_ through Pearson (r = −0.596, *p* = 0.032) and Spearman (*ρ* = −0.676, *p* = 0.011) analyses (Table 9, Entry 6, Figure 6D). Also, TPC displayed a significant positive correlation with FRAP as evident by both Pearson (r = 0.571, *p* = 0.041) and Spearman (*ρ* = 0.652, *p* = 0.016) analyses (Table 9, Entry 7, Figure 6E). Contrary to these observations, Pearson and Spearman analyses showed that TFC has comparatively weak and statistically non-significant correlations with RA concentration, TPC, IC_50_, and FRAP (Table 9, Entry 2,5,8, and 9) with the full dataset, though a moderate trend with IC_50_ was apparent. Besides these correlations, the two antioxidant parameters IC_50_ and FRAP showed a strong negative correlation as evident from both Pearson (r = −0.777, *p* = 0.002) and Spearman (*ρ* = −0.806, *p* = 0.001) analyses (Table 9, Entry 10, Figure 6F). Across both antioxidant endpoints, RA concentration showed stronger associations than TPC, with higher coefficient magnitudes and lower *p*-values. The same ordering was observed with Spearman analysis, suggesting that the difference is not an artefact of distributional assumptions (Table 9, Entry 3 and 4 vs. 6 and 7). Overall, these observations indicated statistically significant correlation among RA concentration, TPC, and antioxidant behavior, underscoring RA as an important antioxidant marker, while TFC demonstrated a statistically non-significant weaker association. As a control to account for commercial samples with unverified origins, the correlation analysis was repeated with the nine field-collected, voucher-authenticated samples. For this subset, all the parameters under study showed approximately normal distribution (Shapiro–Wilk, *p* > 0.05). This confirmed that all core associations were retained or even strengthened within this fully traceable subset (RA vs. TPC: r = 0.762, *p* = 0.017; RA vs. IC_50_: r = −0.756, *p* = 0.019; RA vs. FRAP: r = 0.963, *p* < 0.001; TPC vs. IC_50_: r = −0.746, *p* = 0.021; TPC vs. FRAP: r = 0.719, *p* = 0.029; IC_50_ vs. FRAP: r = −0.768, *p* = 0.016), suggesting that these findings reflect genuine botanical trends.

### 2.9. Geo-Climatic Variability and Varietal Effects on Phytochemical and Antioxidant Parameters

After finding statistically significant correlations between phytochemical parameters and antioxidant effects, geo-climatic variability and varietal effects were investigated. PREs included field samples from diverse geographical regions and commercially obtained samples. Furthermore, a few of this study’s samples are of the Krishna tulsi variety. As TPC, TFC, IC_50_, and FRAP displayed approximate normal distribution as per the Shapiro–Wilk analysis, one-way ANOVA was performed for these parameters. Considering the deviation from normal distribution for RA concentration, the geographical association for RA concentration was checked with the Kruskal–Wallis (KW) analysis. The KW *p*-value for RA concentration is 0.0332 < 0.05, indicating a statistically significant association (Table 10, Entry 1). For TPC, TFC, IC_50_, and FRAP, one-way ANOVA showed *p*-values of 0.039, 0.078, 0.012, and 0.017, respectively, demonstrating significant association for TPC, IC_50_, and FRAP but not for TFC (Table 10, Entries 2–5). Interestingly, effect size calculations showed large associations (η^2^/*ε*^2^ = 0.61–0.87) across all five parameters, including TFC (η^2^ = 0.61), despite its ANOVA *p* > 0.05; consistent with limited statistical power at *n* = 2–3 per zone rather than a true absence of geo-climatic influence. These findings are further reinforced by the independent chemometric clustering presented in Section 2.10.

**Figure 7:**
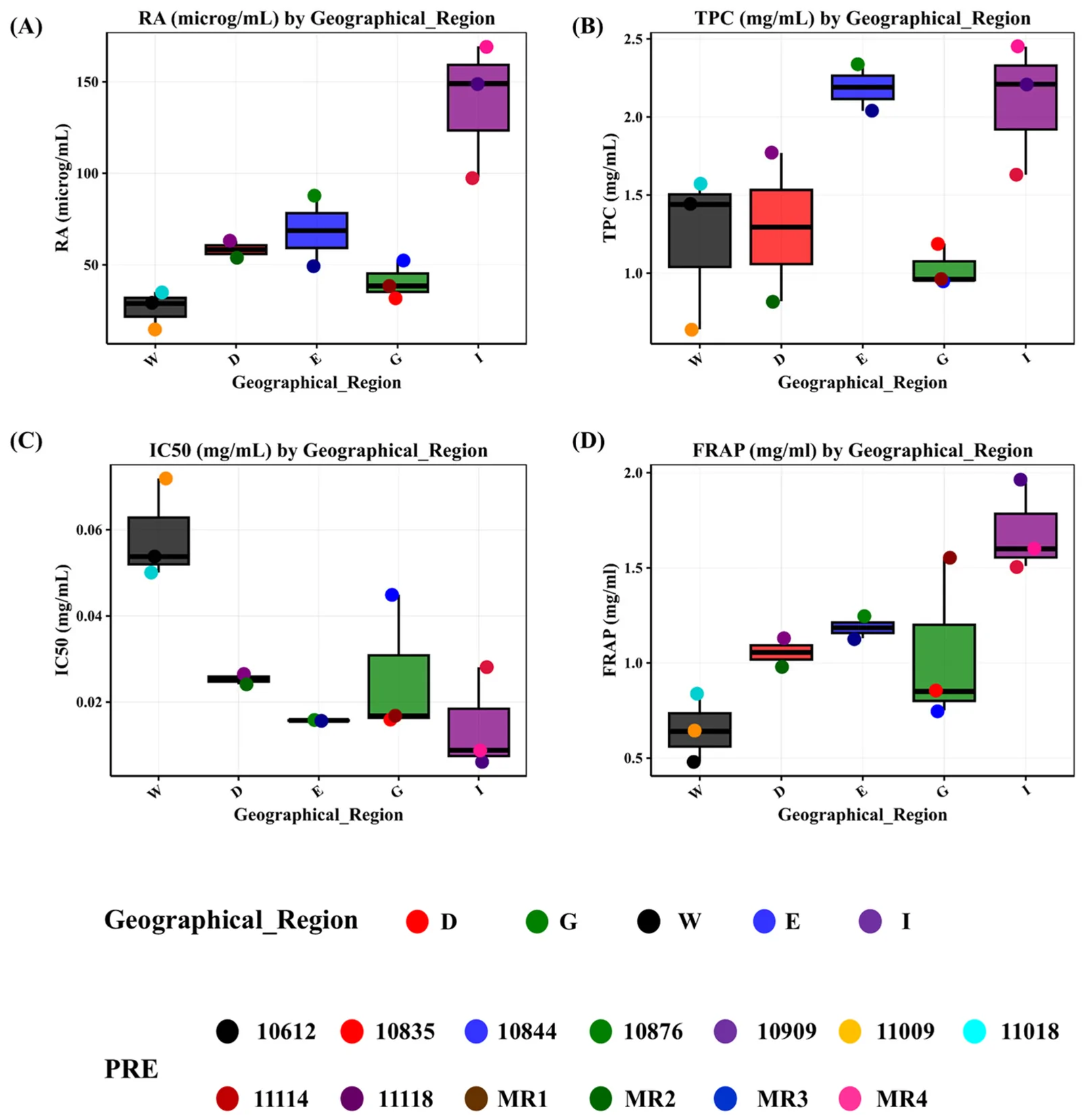
Box plot representation of geo-climatic variability in (**A**) RA concentration (µg/mL), (**B**) TPC (mg GAE/mL), (**C**) IC_50_ (mg/mL), and (**D**) FRAP (mg TE/mL) among the PREs. Box fill colors indicate geo-climatic zones; dots represent individual PREs. **Abbreviations:** RA, rosmarinic acid; TPC, total phenolic content; GAE, gallic acid equivalent; IC_50_, half-maximal inhibitory concentration; FRAP, ferric reducing antioxidant power; TE, Trolox equivalent; PREs, pharmacopeia-relevant extracts; W, Western Ghats; E, Eastern Ghats; D, Deccan; G, Gangetic Plain; I, Indus Plain/Semi-Arid.

The geo-climatic trends of RA concentration, TPC, IC_50_, and FRAP are visually represented by box plot analysis in Figure 7. The box plots showed noticeable inter-geo-climatic variability for RA concentration, TPC, IC_50_, and FRAP. PREs originating from the Indus Plain/Semi-Arid region (I) demonstrated comparatively higher RA concentration and stronger antioxidant-associated behavior, while Western Ghats (W) showed the corresponding lowest values.

Nevertheless, varietal differences for the Krishna variety were statistically non-significant across all the investigated phytochemical and antioxidant parameters, with *p* > 0.05 (Table 10, Entry 6–10).

Together, these results suggested that geographical and associated environmental factors may contribute more significantly toward phytochemical variability than the varietal classifications under study. Effect sizes were calculated to evaluate these associations independent of sample size (Table 10). Geo-climatic zone demonstrated large effects (η^2^/ε^2^ = 0.61–0.87), whereas variety showed negligible to small effects (η^2^/ε^2^ = 0.00–0.03).

### 2.10. Chemometric Evaluation

**Figure 8:**
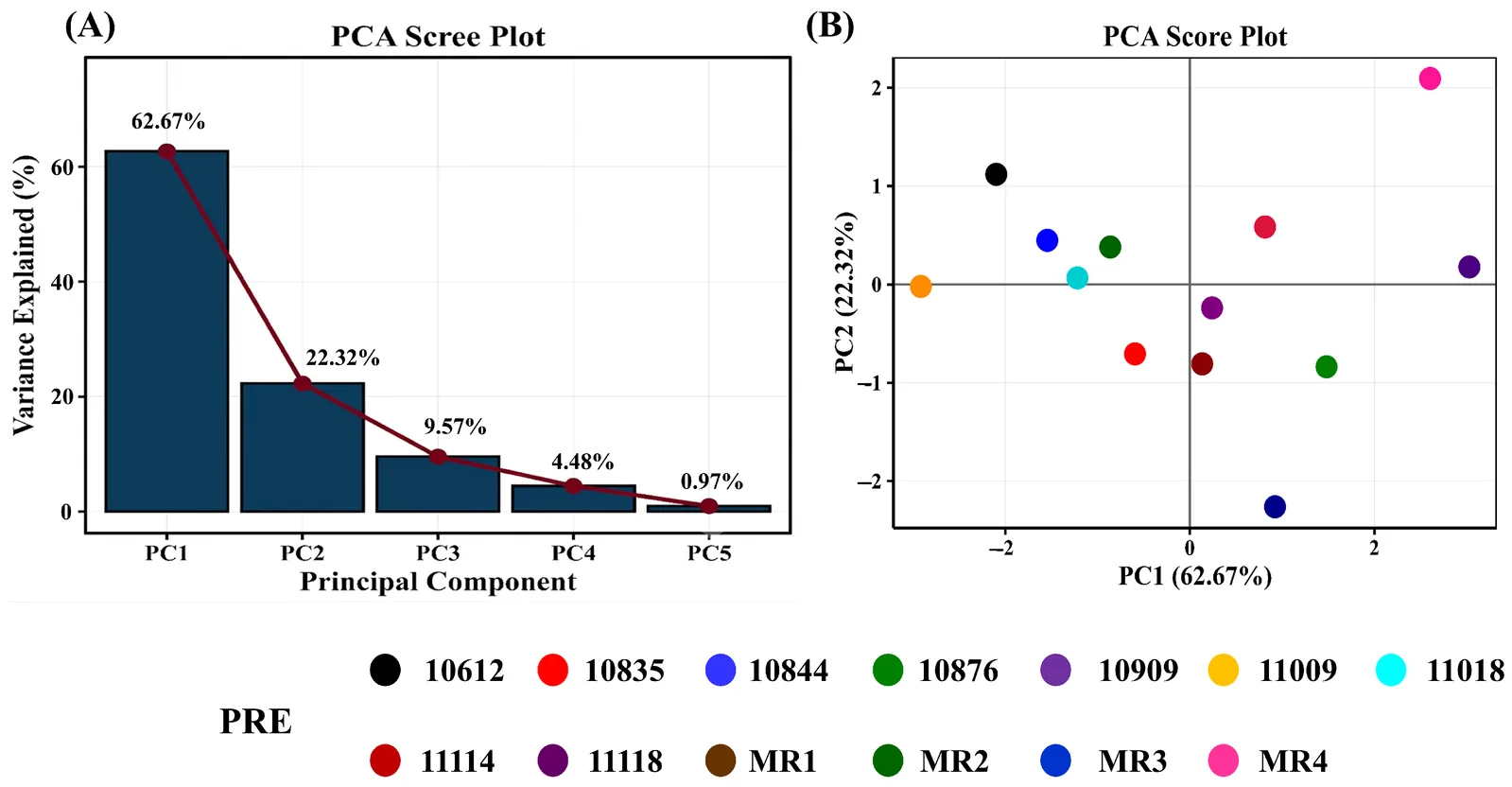
Principal Component Analysis (PCA) of Pharmacopeia-Relevant Extracts (PREs). (**A**) PCA scree plot showing variance of principal components. (**B**) PCA score plot of PREs based on phytochemical and antioxidant parameters.

Chemometric analyses, e.g., Principal Component Analysis (PCA), Hierarchical Cluster Analysis (HCA), dendrogram analysis, and heatmap generation, indicated observable clustering among the investigated PREs. The PCA scree plot, depicting the percentage variance explained by each principal component, showed that PC1 (Dimension 1) and PC2 (Dimension 2) together explained 85.0% of the total variance, with the contribution of PC1 being 62.67% and that of PC2 being 22.32% (Figure 8A). The PCA score plot manifested appreciable clustering tendencies among several PREs (Figure 8B). Samples with higher RA concentration, TPC, and FRAP activity and lower IC_50_ values clustered toward the positive side of PC1. 11118 and MR4 showed comparatively close clustering behavior. Conversely, 10612 and 11009, with comparatively lower RA concentration and lower antioxidant effects, were distributed toward the negative side of PC1.

**Figure 9:**
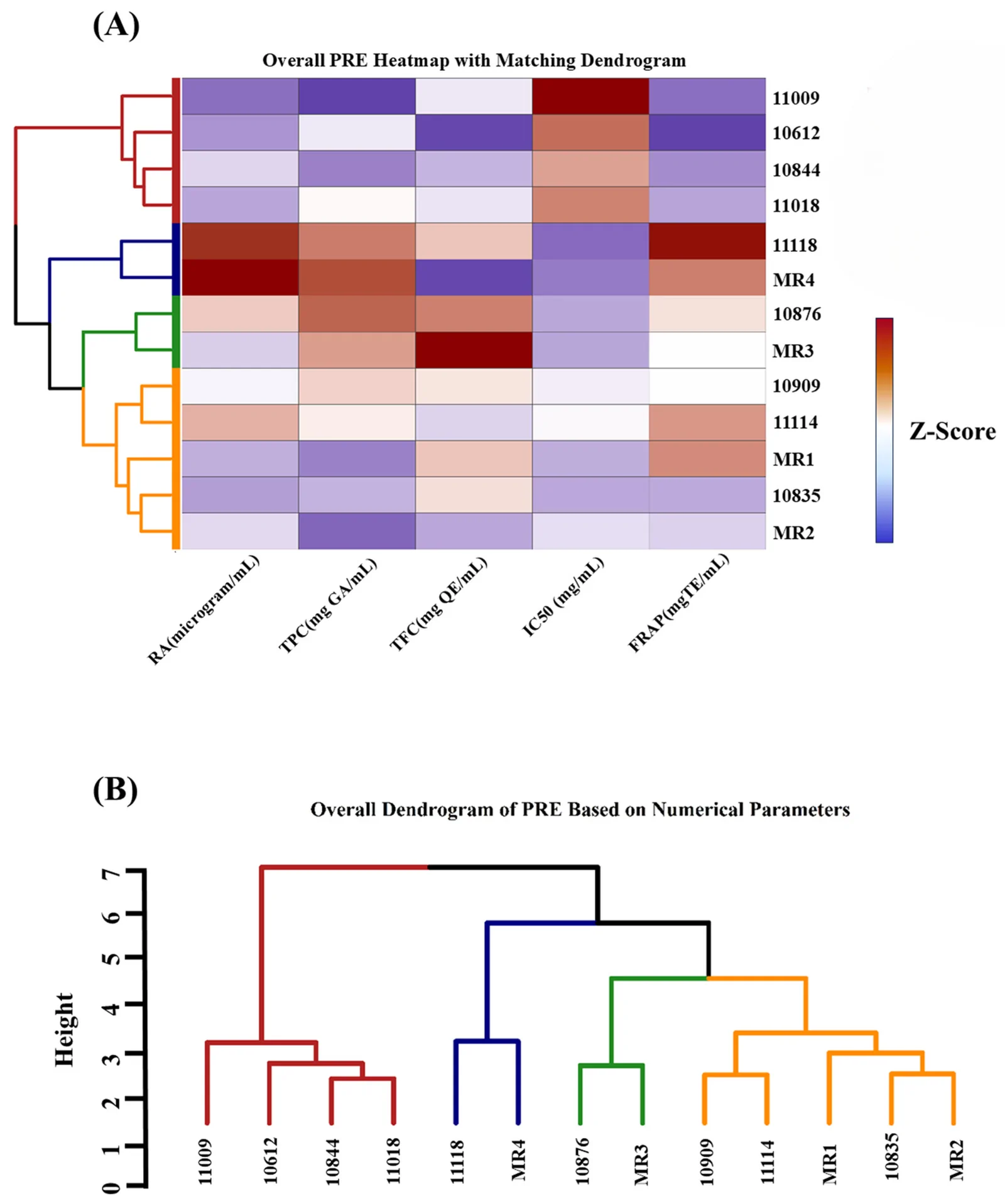
Chemometric clustering analysis of PREs based on RA concentration, TPC, TFC, IC_50_, and FRAP activity. (**A**) Heatmap with hierarchical clustering among PREs. (**B**) Hierarchical cluster dendrogram relationships among PREs. **Abbreviations:** PREs, pharmacopeia-relevant extracts; RA, rosmarinic acid; TPC, total phenolic content; TFC, total flavonoid content; IC_50_, half-maximal inhibitory concentration; FRAP, ferric reducing antioxidant power.

Heatmap data showed clustering patterns among PREs with high RA concentration, TPC, and FRAP activity, together with lower IC_50_ values, while inconsistent clustering was seen for TFC with respect to the antioxidant behaviors (Figure 9A). The heatmap also showed geography-dependent clustering behavior for RA concentration. The hierarchical cluster dendrogram analysis showed close hierarchical relationships among several geographically related PREs (Figure 9B). For example, two of the three Indus Plain/Semi-Arid region (I) samples (11118 and MR4) clustered closely, fueled by their high RA concentration, TPC, and strong antioxidant effects. Most interestingly, several market samples clustered closely with their field samples from the same geographic region (Figure 9B).

## 3. Discussion

Several prior studies on OT and *Ocimum* species have reported antioxidant activity, chromatographic fingerprinting, metabolomic characterization, and chemometric clustering [26,37]. However, many previous investigations restricted themselves to general metabolite-level assessment and often dealt with extremely limited geographic sampling [39]. Previous studies have performed in-depth multi-marker metabolomic studies [39,43]. However, unlike those high-end metabolomic studies, which primarily employed tandem mass spectrometry, the present study not only incorporated but also integrated diverse and orthogonal analytical techniques, e.g., localization of the antioxidant-associated constituent RA via HPTLC-DPPH assay, quantification of RA through HPTLC, and quantification of different phytochemical entities (e.g., TPC and TFC), to functionally bridge them together with their antioxidant effects as demonstrated through DPPH scavenging analysis and FRAP in the bulk extract. However, arguably the most important aspect of the present work is the investigation of clinically relevant extracts rather than laboratory-made customized extracts, which often have negligible translational relevance.

In the present study, RA demonstrated prominent DPPH-responsive antioxidant localization on HPTLC plates. However, UA did not show detectable antioxidant activity under the same conditions. Furthermore, since several unidentified bands were detected in the HPTLC chromatograms, including some bands with antioxidant effects as detected by HPTLC-DPPH bioautography, further investigation of these unidentified bands is warranted in future studies. However, comprehensive identification of every band detected by HPTLC would require elution of individual bands from the plate, followed by direct MS/MS analysis coupled with ion mobility spectrometry, which was beyond the scope of the targeted analytical validation approach adopted in this pharmacopeia-based study; such untargeted metabolite profiling is noted as a direction for future work. This study’s observation suggests that, unlike UA, RA significantly contributes to the antioxidant properties shown by all PREs. Chemically, these antioxidant properties likely originate from the polyphenolic nature of RA. This observation supports the previously reported antioxidant associations of RA-rich *Ocimum* extracts. However, most such antioxidant studies principally deal with the bulk antioxidant effects of the extracts [30,35].

A recent review on the phytochemistry of OT also noted RA as an important antioxidant-related constituent, along with UA. The review found no significant toxic effects associated with RA, whereas UA is reported to show hepatotoxicity [44]. This difference also supports the preference for RA over UA as a standardization marker. Through its catechol group, RA scavenges free radicals and binds metal ions such as Fe^2+^ and Cu^2+^. It was also reported that RA enhances the body’s antioxidant defense through the Nrf2 pathway [45]. These results are essentially in agreement with the direct antioxidant activity of RA observed in the HPTLC-DPPH bioautography in the present study. Therefore, the antioxidant activity observed here, along with the reported safety profile of RA, provides a scientific basis for selecting RA over UA as a standardization marker in pharmacopeia-relevant extracts intended for clinical use.

Results of the molecular docking study (Section 2.2, Table 1) parallel with previous in silico studies on RA, wherein it shows favorable binding with anticancer-linked receptors MARK4, FAK, and HSP90AA1 [45]. This reported study and this study’s in silico findings suggest that RA may act as a multi-target bioactive compound. The reported antioxidant, anti-inflammatory, anticancer, and cognitive-protective activities of RA in OT also correspond well with the activity classes examined in the present docking study [44].

The docking results provided additional, yet purely theoretical, support for selecting RA over UA. RA consistently showed stronger binding affinities than UA across all seven evaluated protein targets (Table 1). However, the docking analysis was purely computational and was not experimentally validated. Therefore, the docking results were considered supportive rather than definitive evidence of biological activity.

The initial fingerprinting and marker identification study showed that eugenol could not be conclusively detected by HPTLC in PREs, whereas it was readily detected simultaneously in the pentane and dichloromethane extracts of the plant material. This is consistent with the role of solvent nature and polarity on metabolic partitioning.

Considerable variability in RA concentration, TPC, TFC, IC_50_ values, and FRAP has been observed in the present study in accordance with previously reported studies [37].

Statistically significant positive correlation between RA concentration, TPC, and FRAP, and negative association with DPPH IC_50_ values, suggests substantial contribution of RA concentration and TPC toward the antioxidant profiles of the PREs under study. Although RA concentration is technically a part of TPC, notably RA concentration correlated stronger with antioxidant parameters compared to TPC, indicating RA concentration tracks the antioxidant behavior more closely than TPC within these PREs under this present study. However, TFC showed statistically non-significant correlations with the antioxidant parameters DPPH-IC_50_ and FRAP, indicating that the antioxidant properties may be more strongly associated with specific phenolic components, particularly RA, rather than TFC, at least under the present experimental conditions (Table 9). However, a modest inverse trend between TFC and IC_50_ was apparent. On a molar basis, TFC (2.08–11.90 μmol/mL QE) and TPC (3.76–14.40 μmol/mL GAE) covered a comparable, overlapping range, indicating that TFC content was not disproportionately low relative to TPC. TFC’s %RSD over the dataset (44.9%) was also higher than that of TPC (39.2%) and FRAP (38.5%), ruling out low variability as an explanation. Furthermore, Monte Carlo permutation testing of all ten pairwise correlations showed that these four TFC-associated correlations remained appreciably non-significant (*p* = 0.21–0.67). However, the remaining six stayed significant, including several with even lower *p*-values. These findings suggest that the weak association of TFC with antioxidant activity is not due to its low content or a statistical artifact. Instead, it may reflect assay-specific sensitivity or compositional independence from the RA-driven antioxidant signal. The box plot clearly shows that the samples from the Indus Plain/Semi-Arid region differ considerably from the Western Ghats samples with respect to RA concentration, TPC, DPPH IC_50_ values, and FRAP values, as evidenced by their distinct and diagonally separated clustering patterns (Figure 7A–D).

Chemometric analyses consisting of PCA clustering, heatmap, and dendrogram analysis demonstrated distinguishable phytochemical-antioxidant patterns among PREs. Comparable chemometric distinction among *Ocimum* species and cultivars of OT has already been reported [37,39]. Interestingly, several commercially obtained samples demonstrated clustering similarity with field samples (e.g., MR4 with 11118, MR3 with 10876, and MR1 with 10835) originating from corresponding geo-climatic regions, despite the absence of direct sourcing traceability for those commercial samples (Figure 9B).

Nevertheless, several limitations remain within the present investigation. Firstly, the antioxidant assays employed in this study are primarily chemical in nature and do not readily translate into direct biological inference without further biological validation studies. However, it is believed that the present investigation may lay the foundation for future biological studies involving extracts possessing diverse phytochemical characteristics. Similarly, the molecular docking results are exploratory in nature and should not be interpreted as evidence of actual biological target engagement or in vivo activity. Another important factor limiting the interpretation of the present study is that, despite statistically significant associations, the observed geographical effects may have been partially entangled with seasonal, environmental, agricultural, and post-harvest interventions. Such entanglement could not be addressed effectively due to the inherent nature of this study, particularly the inclusion of commercial samples with unknown collection and handling histories, alongside relatively small sample sizes within individual regional subsets. To address this possible issue, the correlation analysis was repeated using only the nine voucher-authenticated samples, excluding commercially sourced extracts (Section 2.8); the six statistically significant correlations in Table 9 not only survived but occasionally strengthened in this fully traceable subset, supporting the conclusion that the observed relationships reflect inherent botanical trends rather than artifacts of commercial sample inclusion. However, the clustering of commercial samples together with field samples originating from similar geographical regions highlights meaningful observational trends that point toward potential geographic influences, providing valuable preliminary insights that lay the groundwork for future targeted investigations. The five geo-climatic zones’ samples span a wide geographic range across India, with the two most distant sampling sites separated by around 1600 km, encompassing substantially different climatic and soil conditions. Sample sizes were determined by natural plant availability in the field rather than predetermined targets. To evaluate these trends independently of sample size, effect sizes were calculated, revealing large effects for the geo-climatic zone (η^2^/ε^2^ = 0.61–0.87) and negligible effects for variety (η^2^/ε^2^ = 0.00–0.03). Although RA emerged as a key antioxidant-associated constituent in the investigated PREs, considering the relevance of these preparations in clinical practice, further multidisciplinary investigations are warranted to evaluate how the observed chemical variability influences their biological activity profiles.

Overall, the present study indicates that RA represents a prominent antioxidant-associated phytochemical marker within clinically relevant extracts, contributing to the observed overall antioxidant activity. This study further highlights substantial geo-climatic-associated phytochemical variability and emphasizes the importance of evaluating phytochemical markers through orthogonal confirmation directly within clinically relevant extracts rather than relying solely upon exploratory solvent extracts or untargeted metabolomic profiling.

## 4. Materials and Methods

### 4.1. Plant Material

As mentioned in the pharmacopeia, the aerial parts (whole plant excluding roots) were collected from different geo-climatic regions of India. A total of nine field samples were collected, comprising three samples from the Western Ghats, one from the Eastern Ghats, one from the Deccan region, two from the Gangetic Plain, and two from the Indus Plain/Semi-Arid region. In addition, four commercial samples representing different geographical regions, namely the Eastern Ghats, Deccan, Gangetic Plain, and Indus Plain/Semi-Arid regions, were also included in this study. Overall, this study included three samples from the Western Ghats (W), two samples from the Eastern Ghats (E), three samples from the Indus Plain/Semi-Arid region (I), two samples from the Deccan (D) and three samples from the Gangetic Plain geo-climatic zones. Further, climatic, seasonal, and other environmental conditions were not considered as variables for the present study, since one of this study’s central aims was to assess phytochemical variability under real-world geo-climatic collections including commercial samples. Additionally, such parameters may not closely replicate across these widely different geo-climatic zones, thereby making their independent interpretation difficult considering this study’s scientific framework.

All 13 plant samples, irrespective of their origin (whether field or commercial), were authenticated by J.S., one of the authors of this paper. For commercial samples, the exact cultivation/growing conditions and harvesting details were unavailable. The details of all the samples used for this are provided in Table 11.

Live, healthy specimens were collected along with Global Positioning System (GPS) readings and color photographs for herbarium preparation; field characters (locality, habitat, habit, flower/fruit color, distribution) were recorded at each site. Also, representative *Ocimum tenuiflorum* from natural habitats are shown in Figure S10. Specimens were treated with saturated aqueous mercuric chloride solution, pressed between blotting paper (replaced daily to prevent fungal infection), and dried at 21 ± 2 °C for 60 days. Dried specimens were mounted on herbarium sheets (42 × 29 cm) using the standard technique. Voucher specimens were deposited at the Survey of Medicinal Plants and Research Garden for Homoeopathy (SMPRGH) Herbarium, Centre of Medicinal Plants Research in Homoeopathy, Nilgiri District, Tamil Nadu, under Shashikanth Jale (J.S.) (Herbarium In-Charge). Voucher specimen images with details for all field collections are provided in Supplementary Figure S11a–i.

Field specimens were identified based on morphological characters and taxonomic keys in Gamble’s Flora of the Presidency of Madras [46]. OT was distinguished from the other *Ocimum* species by its four-angled hairy stem, opposite serrate leaves that were often reddish or purple-tinged and pubescent, and purplish to reddish flowers. These characters were compared with those of the larger-leaved *O. basilicum*, the ovate-lanceolate, glabrous to nearly glabrous-leaved *O. americanum*, the woody-based *O. gratissimum*, and the densely white-hairy *O. kilimandscharicum*. Taxonomic identity was further verified by comparison with reference specimens at the Madras Herbarium (MH), Botanical Survey of India, Coimbatore. *Ocimum sanctum* L. is treated as a synonym of OT following Plants of the World Online (POWO). The market samples of OT were identified by organoleptic examination. The leaves were greenish to greenish-brown, with a characteristic aromatic odor and a slightly pungent taste. The Krishna Tulsi variety was identified by its distinctive purple color of the stem, petiole, and inflorescence relative to the green-leaved form. All the samples were identified by one of the authors of this study, Shashikanth Jale (J.S.).

Henceforth, field samples are referred to by their Field No., and Market Sample-1, -2, -3, and -4 will be designated as MR1, MR2, MR3, and MR4 respectively.

### 4.2. Chemicals

Distilled water, methanol, ferric chloride (FeCl_3_), sodium hydroxide (NaOH), hydrochloric acid (HCl), Folin-Ciocalteu reagent (LOBA Chemie, Maharastra, India), sodium carbonate (Na_2_CO_3_), gallic acid, aluminum chloride (AlCl_3_), sodium nitrite (NaNO_2_), quercetin (SRL, Taloja, India), DPPH (2,2-diphenyl-1-picrylhydrazyl) (TCI, Tokyo, Japan), ascorbic acid, sodium acetate (CH_3_COONa), glacial acetic acid (CH_3_COOH) (SRL, Taloja, India), TPTZ (2,4,6-Tripyridyl-S-triazine) (TCI, Tokyo, Japan), Trolox (6-Hydroxy-2,5,7,8-tetramethylchroman-2-carboxylic acid) (TCI, Shanghai, China), rosmarinic acid (Sigma-Aldrich, St. Louis, MO, USA), ursolic acid (TCI, Tokyo, Japan), and eugenol (TCI, Tokyo, Japan) were of AR grade. Pharmacopeia-grade ethanol was procured from Hahnemann Publishing Co. Pvt. Ltd. (Kolkata, India).

### 4.3. Preparation of Hydroalcoholic Extracts

As mentioned in the pharmacopeia, the aerial parts of the plant were used for extract (tincture) preparation. For extractions, 76% (*v*/*v*) aqueous alcohol was used [40]. The moisture content and total dissolved solids were measured as per the methods mentioned in the same pharmacopeia [47,48]. The plant material was macerated at 25 °C for 14 days (i.e., two weeks) following the related pharmacopeial procedure while maintaining the pharmacopeial drug weight-to-solvent volume ratio, 1:10, following the method described in the same pharmacopeia. The resulting mixture was then filtered and stored at around 0–4 °C protected from sunlight until further analysis.

### 4.4. Estimation of Total Phenolic Content (TPC)

The Total Phenolic Content (TPC) was determined using the Folin-Ciocalteu colorimetric assay with slight modifications [49]. Gallic acid was used as the reference standard for the calibration curve. A total of 20 µL of gallic acid solution having concentrations of 0.05, 0.10, 0.20, 0.30, 0.40, and 0.50 mg/mL was taken. To each solution, 1.58 mL of water was added, followed by 100 µL of 2 N Folin-Ciocalteu reagent, and then incubated at 25 °C for 10 min. After that, 300 µL 5% (*w*/*v*) sodium carbonate solution was added and incubated for 45 min at 40 °C. The absorbance of the blue colored solution was measured at 765 nm using a Multiskan Sky 96-well plate reader (ThermoFisher Scientific, Waltham, MA, USA) to obtain a linear regression equation by plotting absorbance vs. gallic acid concentration for the standard calibration curve. Plant extracts diluted ten times were used in place of gallic acid solution for determining the total phenolic content of the samples using the corresponding regression equation as gallic acid equivalent. The results were expressed as milligram gallic acid equivalent per mL of the plant extract (mg GAE/mL). The experiments were carried out in triplicate, and total phenolic content was reported as mean ± standard deviation.

### 4.5. Estimation of Total Flavonoid Content (TFC)

The aluminum chloride (AlCl_3_) colorimetric assay was used to calculate the Total Flavonoid Content (TFC) [50]. Quercetin was used as the reference standard for the calibration curve. To 0.5 mL of quercetin solution having concentrations of 0.025, 0.050, 0.075, 0.10, and 0.15 mg/mL, 2.5 mL of distilled water was added. To this, 0.15 mL of 5% (*w*/*v*) aqueous sodium nitrite was added. Following a 5 min incubation period, 0.3 mL of a 10% (*w*/*v*) aqueous aluminum chloride (AlCl_3_) solution was added, and the mixture was allowed to stand for an additional 5 min. Then, 0.55 mL of distilled water and 1 mL of 1 M sodium acetate were added to the reaction mixture. The absorbance was measured at 415 nm using a Multiskan Sky 96-well plate reader (ThermoFisher Scientific), and the linear regression equation was obtained by plotting absorbance vs. quercetin concentration for the standard calibration curve. Plant extracts diluted ten times were used in place of quercetin solution for determining the total flavonoid content of the samples using the corresponding regression equation and expressed as mg quercetin equivalent/mL (mg QE/mL). The experiments were carried out in triplicate, and total flavonoid content was reported as mean ± standard deviation.

### 4.6. DPPH (2,2-Diphenyl-1-picrylhydrazyl) Radical Scavenging Assay and IC_50_ Determination

The free radical scavenging capacity of the plant extracts was determined using the DPPH assay. It was found that 1 mL of the plant extracts almost quantitatively inhibited 1 mL of 0.2 mM DPPH solution in 95% (*v*/*v*) ethanol. Therefore, the IC_50_ value for such inhibition was determined. For this, 1 mL of 10-, 20-, 40-, 80-, 160-, 320-, 640-, 1280-, 2560-, 5120-, and 10,240-times diluted plant extracts (using the same solvent system as the extract) was treated with 1 mL of 0.2 mM DPPH solution and kept in the dark for 30 min at 25 °C. The concentration of the diluted plant extracts in the reaction mixture was determined from the total solids content of the extracts. Then, the absorbance of the untreated 0.2 mM DPPH solution and the treated DPPH solutions with all dilutions was measured at 517 nm using a Multiskan Sky 96-well plate reader (Thermo Fisher Scientific).

The IC_50_ values were determined by employing nonlinear regression via a Four-Parameter Logistic (4PL) sigmoidal dose–response model implemented through a Python 3.14-based analysis script using SciPy 1.17. The experiments were carried out in triplicate, and IC_50_ values were reported as mean ± standard deviation. Ascorbic acid was used as the reference standard.

### 4.7. Ferric Reducing Antioxidant Power (FRAP) Assay

The Ferric Reducing Antioxidant Power (FRAP) assay was carried out using freshly prepared FRAP reagent consisting of 300 mM acetate buffer, 10 mM TPTZ solution prepared in 40 mM hydrochloric acid, and 20 mM ferric chloride solution in distilled water mixed in a ratio of 10:1:1 (*v*/*v*/*v*) [51]. The reagent mixture was incubated at 37 °C for 10 min prior to use. For the assay, 3 mL of the FRAP reagent was mixed with 0.1 mL of the sample solution and incubated at 37 °C for 10 min. The absorbance was then measured at 593 nm using a 96-well plate reader (Thermo Fisher Scientific). Trolox was used as the reference standard for the linear calibration curve, and standard solutions in the concentration range of 0.8065–8.0645 mg/L in the final reaction mixture were prepared. Plant extracts diluted ten times were used in place of the Trolox solution for the determination of antioxidant capacity. The FRAP values of the samples were calculated using the corresponding linear regression equation and expressed as Trolox equivalents (in mg/mL). The experiments were carried out in triplicate, and FRAP was reported as mean ± standard deviation.

### 4.8. Procedure for High-Performance Thin-Layer Chromatography

#### 4.8.1. Preparation of Standard Stock Solution

The reference standard, RA, was obtained from Sigma-Aldrich (St. Louis, MO, USA), while UA and eugenol were obtained from TCI (Tokyo, Japan). An accurately measured quantity of 1 mg of each standard was transferred into separate 10 mL volumetric flasks and diluted to the mark with methanol. A 1 mL aliquot of this stock solution, having a concentration of 100 µg/mL, was further diluted to 2 mL with methanol, resulting in working standard solutions with a concentration of 0.05 mg/mL. These stock and working standard solutions were preserved at 4 °C, and any unused solution was discarded the next day.

#### 4.8.2. Instrumentation and Chromatographic Conditions and Procedures of HPTLC

This study employed the Camag HPTLC system (Muttenz, Switzerland), consisting of a Linomat 5 semi-automatic sample applicator equipped with a 100 µL Hamilton syringe (Hamilton Bonaduz AG, Bonaduz, Switzerland), TLC Scanner 4, a twin-trough developing chamber (20 × 10 cm), a UV cabinet featuring a dual-wavelength UV lamp, Plate Heater 3, and TLC Visualizer 3. The operation of the Linomat 5 sample applicator and TLC Scanner 4 was facilitated by visionCATS software, while TLC Visualizer 3 was operated through visionCATS (Version 3.1) for photo-documentation purposes. The spots were detected under 254 nm, 366 nm, and white light before and after derivatization (as applicable). Calculations were performed using Microsoft Excel 2007 software.

The samples were applied as bands with a bandwidth of 8 mm using a 100 µL Hamilton syringe on precoated silica gel aluminum-backed TLC Plate 60 F_254_ (20 × 10 cm, 100 µm thickness) supplied by E. Merck, Darmstadt, Germany. A uniform application rate of 150 nL/s was employed. Plate development was carried out using a mobile phase consisting of toluene, ethyl acetate, and formic acid in a ratio of 5:4:1 (*v*/*v*/*v*) for RA, hexane and ethyl acetate in a ratio of 4:1 (*v*/*v*) for UA and eugenol after equilibration of the chamber for 20 min at 25 °C. Formic acid (98%) was obtained from SRL (Taloja, India), while methanol, toluene, and ethyl acetate were of AR grade and procured from Merck Life Science Pvt. Ltd. (Mumbai, India).

For densitometric analysis, the Camag TLC Scanner 4 coupled with visionCATS software was utilized. The slit dimensions and scanning speed were maintained at 5 × 0.2 mm and 20 mm/s, respectively. The concentrations were calculated by plotting the intensity of diffused reflected light from the separated compounds against their respective concentrations and applying linear regression analysis. Spectra for the HPTLC method were recorded by scanning the bands over a wavelength range of 220–400 nm at a scanning speed of 100 nm/s, and the spectra were documented. The wavelength showing maximum absorbance was selected as the detection wavelength for the method. RA was quantified at a UV wavelength of 325 nm.

#### 4.8.3. Fingerprinting and Identification of Markers in the Plant Extracts

Different plant extracts from different samples were applied onto a TLC plate, developed according to the prescribed method, and dried at room temperature. The Rf values and colors of the resolved bands were recorded with and without derivatization.

For the detection and subsequent identification of RA, a mobile phase consisting of toluene, ethyl acetate, and formic acid in a ratio of 5:4:1 (*v*/*v*/*v*) was used, while for UA and eugenol, a mobile phase consisting of hexane and ethyl acetate in a ratio of 4:1 (*v*/*v*) was used. In HPTLC, the detection of the marker compounds was done by spectral matching of the spots from the Pharmacopeia-Relevant Extract (PREs), with the reference standards having matched R_f_ values and colors.

##### Anisaldehyde Sulphuric Acid Reagent (ASR) Derivatization

The dried developed plate was immersed in ASR for 5 s and heated at 105 °C for 5 min. The developed colors were photographed under white light. Changes in the color of the spots were noted along with the appearance of new spots.

##### Ferric Chloride Derivatization

The dried developed plate was immersed in 1% (*w*/*v*) ferric chloride solution in 0.5 M hydrochloric acid for 2 s and kept at 25 °C for 10 min for drying. The developed colors were photographed under white light. Changes in the color of the spots were noted along with the appearance of new spots, chiefly for phenolics.

##### NP/PEG Derivatization

The dried developed plate was first immersed in 1% diphenylboric acid aminoethyl ester in acetone (NP reagent) for 2 s and kept at 25 °C for 10 min for drying. Then, the plate was immersed in PEG reagent (5% polyethylene glycol 4000 in ethanol) for 2 s and again dried at room temperature. After derivatization, the plate was photographed under 366 nm UV light, and the fluorescent bands were noted, chiefly for phenolics and flavonoids.

##### DPPH Bioautography

The dried developed plate was immersed in 0.2% (*w*/*v*) DPPH in absolute ethanol for 2 s. Then, the plate was kept at 25 °C for 30 min in the dark. After that, the plate was photographed under white light. The bleaching of the purple DPPH color at the spots was noted for antioxidant chemicals.

#### 4.8.4. Quantification of RA

The developed HPTLC method was validated for specificity, linearity, sensitivity, accuracy, precision, repeatability, and robustness in accordance with the International Council for Harmonisation (ICH) guidelines Q2(R1). The linearity of RA under HPTLC conditions was determined by applying different volumes of the same standard working solution of RA over the concentration range of 250–600 ng/band, and the calibration curve was constructed by plotting total absorbance against concentration at 325 nm in five replicates (*n* = 5). The calibration data were subjected to least-squares linear regression analysis. The sensitivity of the method was assessed by determining the Limit Of Detection (LOD) and Limit Of Quantification (LOQ) of RA. The LOD and LOQ values were calculated using Microsoft Excel through regression analysis based on the Standard Deviation (SD) of the y-intercept and the Slope (S) of the calibration curve using the equations LOD = 3.3 × (SD/S) and LOQ = 10 × (SD/S).

The precision of the method was evaluated by intraday (*n* = 3) and interday (*n* = 3) precision studies. Even though there are reports of using the standard solution instead of the extract itself for the study, the plant extract itself was used for the precision study to account for possible matrix effects. The accuracy study was performed via recovery, wherein the plant extracts were spiked with a known amount of RA, and the percentage recovery was calculated [52,53].

The specificity of the analysis was checked by comparing the R_f_ value, UV spectra, and peak purity of RA at different times and dates. The peak purity of RA in the sample was checked by comparing its UV–Vis spectra with those of standard RA. The robustness of the study was determined by deliberate small alterations in saturation time, elution length, and, most importantly, the composition of the mobile phase.

### 4.9. Purification of RA

A total of 500 mL PRE of 11118 was defatted using three 100 mL portions of n-hexane, and the solvent from the defatted extract was removed using a rotary evaporator with the water bath temperature not exceeding 40 °C. The dried residue was dissolved in methanol and filtered through a silica bed. RA was first semi-purified using 35% methanol in chloroform (*v*/*v*) via column chromatography, followed by purification by preparative TLC using the same solvent system as used in HPTLC. The overall yield of the material was not measured. The structure of the compound was further confirmed by HRMS (**−ESI**), ^1^H NMR, and ^13^C NMR.

### 4.10. Statistical Analysis

Statistical analyses and corresponding visual representations (e.g., heatmap, dendrogram, PCA scree plot, correlation diagrams) were performed using the R programming environment (version 4.6.0). The dataset was imported from CSV format.

For each sample, a unique sample number was assigned as the sample identifier. For field samples, it was the same as the Field No., while for market samples, they were designated as MR(n) ((n) = 1–4). Variables were automatically classified into Numerical Measurements (NM) and Non-Numerical Metadata (NNM). Numerical variables were converted into numeric format, and categorical variables were treated as factors. Firstly, normality of numerical variables was evaluated using the Shapiro–Wilk test. Correlation analysis among numerical variables was carried out using both Pearson’s and Spearman’s correlation coefficients, without adjustment for multiple comparisons, as a specific, fixed group of related chemical parameters was tested, and every result was reported regardless of significance, and statistical significance was considered at *p* < 0.05. Pairwise correlations (Table 9) were additionally cross-checked using Monte Carlo permutation testing (100,000 resamples per pair), applied uniformly across all ten parameter pairs.

Correlation coefficients (r) were interpreted as follows:

(a) |r| ≥ 0.90: very strong correlation;
(b) |r| = 0.70–0.89: strong correlation;
(c) |r| = 0.50–0.69: moderate correlation;
(d) |r| = 0.30–0.49: weak correlation;
(e) |r| < 0.30: very weak correlation.

This was done to evaluate correlations under both parametric and non-parametric conditions, thereby improving the robustness and reliability of the interpretation. Correlation matrices were further visualized under standardized conditions, simultaneously using heatmaps. Group differences between non-numerical (categorical) and numerical variables were analyzed using both one-way ANOVA (normally distributed variables) and by Kruskal–Wallis tests (non-normally distributed variables). Sample sizes per zone ranged from 2 to 3, reflecting a design that prioritized geo-climatic and commercial diversity over balanced group sizes; all collected samples were retained rather than subsampled for parity. Effect sizes (η^2^ for ANOVA, ε^2^ for Kruskal–Wallis) were additionally calculated to quantify the magnitude of association independent of sample size and are reported alongside *p*-values in Table 10. One-way ANOVA, followed by Tukey’s HSD post hoc test (α= 0.05), was used to compare RA, TPC, TFC, IC_50_, and FRAP values across the 13 PREs. Statistically significant differences were indicated by different superscript letters in Table 2, Table 4, Table 5 and Table 6.

Data visualization was done with boxplots with jittered sample points, bar plots, and two-dimensional scatter plots with regression lines. Principal Component Analysis (PCA) was performed by employing standardized numerical variables to estimate variance distribution and sample clustering. Furthermore, hierarchical clustering analysis was executed using Euclidean distance and Ward’s D2 method, and the outcomes were graphically represented as scaled dendrograms.

### 4.11. Molecular Docking Study

In the context of the molecular docking study, the 2D structures of the bioactive compounds, RA and UA, were drawn using ChemDraw version 3.0 and converted into energy-minimized 3D structures with Chem3D version 3.0. LeDock (win32) is a user-friendly, accurate, and semi-flexible Graphical User Interface (GUI) docking tool, where proteins are considered rigid and ligand structures bind flexibly to the active site. Seven different types of proteins from various targets were selected from the ‘LePro’ module of LeDock (win32), and grid box parameters were automatically selected to cover the geometric pattern occupied by the prototype in-bound ligand in the crystal structures of the selected Protein Data Bank (PDB), with an RMSD cut-off range of 1.0 to minimize redundancy in docking poses. The docking was then initiated. The final docking results, including docking scores (kcal/mol), were reported, and the docked poses of the compounds were visually inspected using PyMOL (version 3.1.9), while 2D binding interaction plots were generated by Discovery Studio Visualizer version 3.0.

## 5. Conclusions

This study demonstrated substantial phytochemical and antioxidant-associated variability among PREs of OT collected from five different geo-climatic regions and commercial sources across India. HPTLC fingerprinting consistently identified RA and UA across all PREs under study. However, the HPTLC-DPPH bioautography assay revealed prominent antioxidant-activity localization for RA alongside other unidentified spots, whereas UA exhibited no detectable activity. Moreover, although eugenol was readily detectable in non-polar extracts, it could not be conclusively detected in the PREs prepared from the exact same raw drug, highlighting the significant influence of extraction solvent polarity on phytochemical partitioning. Together with exploratory docking exercises, which also pointed toward a comparatively higher antioxidant-associated and receptor-binding potential, these findings led to the selection of RA as the major marker compound for subsequent quantitative and chemometric investigations.

Quantitative HPTLC analysis on 13 PREs showed considerable heterogeneity in RA concentration among the PREs. Positive correlations among RA concentration, TPC, and FRAP activity, along with an inverse association with DPPH-based IC_50_ values, suggested a potential role of RA and overall phenolics toward the antioxidant-associated behavior of the investigated PREs under the present experimental conditions. However, notably, RA concentration emerged as the parameter more strongly associated with the antioxidant behavior of the investigated PREs, exceeding TPC in both magnitude and statistical strength. These associations remained robust when the analysis was restricted to the field-collected, voucher-authenticated samples alone, excluding all commercially sourced extracts, reinforcing that the observed relationships reflect genuine botanical trends. In contrast, TFC demonstrated a comparatively weaker and statistically non-significant association with antioxidant parameters with the full dataset.

Geo-climatic variations demonstrated statistically significant variability in RA concentration, TPC, IC_50_, and FRAP among the investigated PREs. Conversely, varietal differences were statistically non-significant across these metrics. This distinction was further supported by effect size estimation, which showed consistently large magnitudes for geo-climatic associations compared to negligible values for varietal associations. Within the constraints of this sample distribution, this suggests that geo-climatic differences may contribute more substantially toward phytochemical and antioxidant differences than the phenotypic classifications investigated under the present study conditions, providing a foundational framework for exploration in future studies with expanded and more balanced sample sizes.

Distinguishable clustering tendencies among PREs were observed through chemometric analyses, including PCA, hierarchical clustering, dendrogram, and heatmap analyses. Notably, although the commercial samples lacked direct cultivation traceability and were categorized solely by their region of procurement, they exhibited clustering similarities with authenticated field samples from those corresponding regions. This observation raises the possibility that these market samples were locally grown, presenting a trend that warrants systematic investigation in future studies.

Ultimately, this study underscores the importance of integrating antioxidant-associated localization, overall antioxidant evaluation, orthogonal phytochemical characterization, and chemometric analyses directly within clinically and pharmacopeia-relevant extracts for improved phytochemical assessment and herbal drug sourcing, production, and standardization.

## Figures and Tables

**Table 1:** Comparative molecular docking analysis of Rosmarinic Acid (RA) and Ursolic Acid (UA) with selected therapeutically relevant protein targets.

Entry	PDB ^1^	Binding Affinity of RA (kcal/mol)	Binding Affinity of UA (kcal/mol)	Associated Target Activity (Theoretical)
1.	3LN1	−6.70	−1.05	Anti-inflammatory
2.	4KFB	−6.60	−1.71	
3.	5IKT	−7.51	−5.01	
4.	1M17	−7.33	−4.81	Anticancer
5.	1O86	−7.18	−5.33	Cardioprotective
6.	2V5Z	−7.66	−3.70	Neuroprotective
7.	3NVY	−8.22	−4.07	Antioxidant

^1^ Protein Data Bank; Rosmarinic Acid (RA); Ursolic Acid (UA).

**Table 2:** Rosmarinic Acid (RA) concentration in Pharmacopeia-Relevant Extracts (PREs) determined by HPTLC densitometric analysis.

Entry	PRE	Geo-Climatic Zone ^2^	Variety ^2^	Rosmarinic Acid Concentration (in µg/mL) [Mean ± SD (% CV)] ^1^
1.	10612	W	NK	28.82 ± 0.29 (1.01%) ^k^
2.	10835	G	K	32.10 ± 0.19 (0.60%) ^j^
3.	10844	G	NK	52.22 ± 0.38 (0.72%) ^f^
4.	10876	E	K	87.68 ± 0.38 (0.43%) ^d^
5.	10909	D	NK	63.06 ± 0.52 (0.82%) ^e^
6.	11009	W	NK	14.54 ± 0.07 (0.45%) ^l^
7.	11018	W	K	35.07 ± 0.23 (0.67%) ^i^
8.	11114	I	K	97.81 ± 0.30 (0.31%) ^c^
9.	11118	I	NK	149.00 ± 2.10 (1.41%) ^b^
10.	MR1	G	NK	38.46 ± 0.90 (2.33%) ^h^
11.	MR2	D	NK	53.61 ± 0.83 (1.54%) ^f^
12.	MR3	E	NK	49.72 ± 0.25 (0.50%) ^g^
13.	MR4	I	NK	169.40 ± 0.58 (0.34%) ^a^

^1^ Values followed by the same superscript letter are not significantly different (one-way ANOVA, followed by Tukey’s HSD post hoc test, *p* < 0.05, *n* = 3). SD, standard deviation; CV, coefficient of variation. ^2^ W, Western Ghats; E, Eastern Ghats; D, Deccan; G, Gangetic Plain; I, Indus Plain/Semi-Arid; K, Krishna; NK, Non-Krishna.

**Table 3:** Linearity regression parameters, correlation coefficients (R and R^2^), Limit Of Detection (LOD), and Limit Of Quantification (LOQ) for Rosmarinic Acid (RA) in High-Performance Thin-Layer Chromatography (HPTLC).

No.	Parameters	Values
1.	Linearity range (ng/band)	250–600
2.	Regression equation	y = 1.616 × 10^−5^x+ 5.998 × 10^−3^
3.	R^2^	0.993
4.	R	0.996
5.	LOD (ng/band)	38.85
6.	LOQ (ng/band)	117.73

**Table 4:** Total Phenolic Content (TPC) and Total Flavonoid Content (TFC) in Pharmacopeia-Relevant Extracts (PREs).

Entry	PRE	Geo-Climatic Zone ^2^	Variety ^2^	TPC (mg GAE/mL) [Mean ± SD] ^1^	TFC (mg QE/mL) [Mean ± SD] ^1^
1.	10612	W	NK	1.44 ± 0.073 ^f^	0.627 ± 0.009 ^i^
2.	10835	G	K	1.19 ± 0.013 ^g^	2.00 ± 0.005 ^d^
3.	10844	G	NK	0.95 ± 0.022 ^h^	1.33 ± 0.023 ^g^
4.	10876	E	K	2.34 ± 0.084 ^ab^	2.64 ± 0.026 ^b^
5.	10909	D	NK	1.77 ± 0.022 ^d^	1.96 ± 0.034 ^d^
6.	11009	W	NK	0.64 ± 0.048 ^i^	1.65 ± 0.022 ^e^
7.	11018	W	K	1.57 ± 0.103 ^ef^	1.62 ± 0.014 ^e^
8.	11114	I	K	1.63 ± 0.033 ^de^	1.52 ± 0.014 ^f^
9.	11118	I	NK	2.21 ± 0.035 ^b^	2.17 ± 0.005 ^c^
10.	MR1	G	NK	0.96 ± 0.043 ^h^	2.17 ± 0.014 ^c^
11.	MR2	D	NK	0.82 ± 0.047 ^h^	1.24 ± 0.020 ^h^
12.	MR3	E	NK	2.04 ± 0.077 ^c^	3.60 ± 0.020 ^a^
13.	MR4	I	NK	2.45 ± 0.008 ^a^	0.635 ± 0.005 ^i^

^1^ Values within a column followed by the same superscript letter are not significantly different (one-way ANOVA, followed by Tukey’s HSD post hoc test, α = 0.05, *n* = 3). SD, standard deviation; GAE, gallic acid equivalent; QE, quercetin equivalent. ^2^ W, Western Ghats; E, Eastern Ghats; D, Deccan; G, Gangetic Plain; I, Indus Plain/Semi-Arid; K, Krishna; NK, Non-Krishna.

**Table 5:** Half-maximal Inhibitory Concentration (IC_50_) values of Pharmacopeia-Relevant Extracts (PREs) in the 2,2-diphenyl-1-picrylhydrazyl (DPPH) radical scavenging assay, using four-parameter logistic (4PL) nonlinear regression analysis.

Entry	PRE	Geo-Climatic Zone ^2^	Variety ^2^	IC_50_ (mg/mL) [Mean ± SD] ^1^
1.	10612	W	NK	0.0538 ± 0.00090 ^b^
2.	10835	G	K	0.0159 ± 0.00005 ^g^
3.	10844	G	NK	0.0449 ± 0.00007 ^d^
4.	10876	E	K	0.0158 ± 0.00031 ^g^
5.	10909	D	NK	0.0265 ± 0.00044 ^ef^
6.	11009	W	NK	0.0719 ± 0.00130 ^a^
7.	11018	W	K	0.0501 ± 0.00096 ^c^
8.	11114	I	K	0.0281 ± 0.00043 ^e^
9.	11118	I	NK	0.0061 ± 0.00150 ^h^
10.	MR1	G	NK	0.0168 ± 0.00153 ^g^
11.	MR2	D	NK	0.0241 ± 0.00210 ^f^
12.	MR3	E	NK	0.0156 ± 0.00028 ^g^
13.	MR4	I	NK	0.0088 ± 0.00045 ^h^

^1^ Values within a column followed by the same superscript letter are not significantly different (one-way ANOVA, followed by Tukey’s HSD post hoc test, α = 0.05, *n* = 3). SD, standard deviation. ^2^ W, Western Ghats; E, Eastern Ghats; D, Deccan; G, Gangetic Plain; I, Indus Plain/Semi-Arid; K, Krishna; NK, Non-Krishna.

**Table 6:** Ferric Reducing Antioxidant Power (FRAP) values of Pharmacopeia-Relevant Extracts (PREs).

Entry	PRE	Geo-Climatic Zone ^2^	Variety ^2^	FRAP (mg TE/mL) [Mean ± SD] ^1^
1.	10612	W	NK	0.48 ± 0.009 ^j^
2.	10835	G	K	0.85 ± 0.025 ^g^
3.	10844	G	NK	0.75 ± 0.037 ^h^
4.	10876	E	K	1.24 ± 0.040 ^d^
5.	10909	D	NK	1.13 ± 0.003 ^e^
6.	11009	W	NK	0.64 ± 0.013 ^i^
7.	11018	W	K	0.83 ± 0.003 ^g^
8.	11114	I	K	1.51 ± 0.035 ^c^
9.	11118	I	NK	1.97 ± 0.033 ^a^
10.	MR1	G	NK	1.55 ± 0.023 ^bc^
11.	MR2	D	NK	0.98 ± 0.01 ^f^
12.	MR3	E	NK	1.13 ± 0.044 ^e^
13.	MR4	I	NK	1.60 ± 0.012 ^b^

^1^ Values within a column followed by the same superscript letter are not significantly different (one-way ANOVA, followed by Tukey’s HSD post hoc test, α = 0.05, *n* = 3). SD, standard deviation; TE, Trolox equivalent. ^2^ W, Western Ghats; E, Eastern Ghats; D, Deccan; G, Gangetic Plain; I, Indus Plain/Semi-Arid; K, Krishna; NK, Non-Krishna.

**Table 7:** Integrated phytochemical content and antioxidant activity rankings of Pharmacopeia-Relevant Extracts (PREs), with post hoc significance groupings ^1^.

Entry	PRE	Geo-Climatic Zone ^2^	Variety ^2^	Mean RA (µg/mL) ^1,3^	Mean TPC (mg GAE/mL) ^1,3^	Mean TFC (mg QE/mL) ^1,3^	Mean IC_50_ (mg/mL) ^1,3^	Mean FRAP (mg TE/mL) ^1,3^
1.	10612	W	NK	28.82 (12) ^k^	1.44 (8) ^f^	0.627 (13) ^i^	0.0538 (12) ^b^	0.48 (13) ^j^
2.	10835	G	K	32.10 (11) ^j^	1.19 (9) ^g^	1.997 (5) ^d^	0.0159 (5) ^g^	0.85 (9) ^g^
3.	10844	G	NK	52.22 (7) ^f^	0.95 (11) ^h^	1.332 (10) ^g^	0.0449 (10) ^d^	0.75 (11) ^h^
4.	10876	E	K	87.68 (4) ^d^	2.34 (2) ^ab^	2.642 (2) ^b^	0.0158 (4) ^g^	1.24 (5) ^d^
5.	10909	D	NK	63.06 (5) ^e^	1.77 (5) ^d^	1.956 (6) ^d^	0.0265 (8) ^ef^	1.13 (6) ^e^
6.	11009	W	NK	14.54 (13) ^l^	0.64 (13) ^i^	1.647 (7) ^e^	0.0719 (13) ^a^	0.64 (12) ^i^
7.	11018	W	K	35.07 (10) ^i^	1.57 (7) ^ef^	1.620 (8) ^e^	0.0501 (11) ^c^	0.83 (10) ^g^
8.	11114	I	K	97.81 (3) ^c^	1.63 (6) ^de^	1.518 (9) ^f^	0.0281 (9) ^e^	1.51 (4) ^c^
9.	11118	I	NK	149.00 (2) ^b^	2.21 (3) ^b^	2.172 (3) ^c^	0.0061 (1) ^h^	1.97 (1) ^a^
10.	MR1	G	NK	38.46 (9) ^h^	0.96 (10) ^h^	2.172 (3) ^c^	0.0168 (6) ^g^	1.55 (3) ^bc^
11.	MR2	D	NK	53.61 (6) ^f^	0.82 (12) ^h^	1.243 (11) ^h^	0.0241 (7) ^f^	0.98 (8) ^f^
12.	MR3	E	NK	49.72 (8) ^g^	2.04 (4) ^c^	3.596 (1) ^a^	0.0156 (3) ^g^	1.13 (6) ^e^
13.	MR4	I	NK	169.40 (1) ^a^	2.45 (1) ^a^	0.635 (12) ^i^	0.0088 (2) ^h^	1.60 (2) ^b^

^1^ Values in parentheses indicate the ranking of the PRE for the corresponding parameter (for IC_50_, rank 1 = lowest value; for all other parameters, rank 1 = highest value). Superscript letters indicate Tukey’s HSD post hoc groupings (one-way ANOVA, α = 0.05, *n* = 3) as reported in Table 2 (RA), Table 4 (TPC, TFC), Table 5 (IC_50_), and Table 6 (FRAP); values within a column sharing the same superscript letter are not significantly different. Letter groupings are independent across columns. ^2^ W, Western Ghats; E, Eastern Ghats; D, Deccan; G, Gangetic Plain; I, Indus Plain/Semi-Arid; K, Krishna; NK, Non-Krishna. ^3^ RA, rosmarinic acid; TPC, total phenolic content; TFC, total flavonoid content; IC_50_, half-maximal inhibitory concentration; FRAP, ferric reducing antioxidant power; GAE, gallic acid equivalent; QE, quercetin equivalent; TE, Trolox equivalent.

**Table 8:** Shapiro–Wilk normality analysis of phytochemical and antioxidant parameters among Pharmacopeia-Relevant Extracts (PREs).

Entry	Parameter ^1^	*W*-Value	*p*-Value	Distribution
1.	RA (µg/mL)	0.855	0.033	Non-normal
2.	TPC (mg GAE/mL)	0.949	0.585	Normal
3.	TFC (mg QE/mL)	0.948	0.568	Normal
4.	IC_50_ (mg/mL)	0.892	0.104	Normal
5.	FRAP (mg TE/mL)	0.967	0.860	Normal

^1^ RA, rosmarinic acid; TPC, total phenolic content; TFC, total flavonoid content; IC_50_, half-maximal inhibitory concentration; FRAP, ferric reducing antioxidant power; GAE, gallic acid equivalent; QE, quercetin equivalent; TE, Trolox equivalent.

**Table 9:** Pearson’s and Spearman’s correlation analysis of phytochemical and antioxidant parameters among Pharmacopeia-Relevant Extracts (PREs).

Entry	Variable 1	Variable 2	Pearson’s r (*p*-Value)	Pearson Interpretation	Spearman’s *ρ* (*p*-Value)	Spearman Interpretation
1.	RA ^a^	TPC ^b^	0.748 (0.003)	Significant—Strong positive correlation	0.692 (0.009)	Significant—Moderate positive correlation
2.	RA ^a^	TFC ^c^	−0.126 (0.681)	Non-significant—Very weak negative correlation	−0.006 (0.986)	Non-significant—Very weak negative correlation
3.	RA ^a^	IC_50_^d^	−0.642 (0.018)	Significant—Moderate negative correlation	−0.648 (0.017)	Significant—Moderate negative correlation
4.	RA ^a^	FRAP ^e^	0.803 (0.001)	Significant—Strong positive correlation	0.806 (0.001)	Significant—Strong positive correlation
5.	TPC ^b^	TFC ^c^	0.242 (0.425)	Non-significant—Very weak positive correlation	0.259 (0.393)	Non-significant—Very weak positive correlation
6.	TPC ^b^	IC_50_^d^	−0.596 (0.032)	Significant—Moderate negative correlation	−0.676 (0.011)	Significant—Moderate negative correlation
7.	TPC ^b^	FRAP ^e^	0.571 (0.041)	Significant—Moderate positive correlation	0.652 (0.016)	Significant—Moderate positive correlation
8.	TFC ^c^	IC_50_^d^	−0.372 (0.211)	Non-significant—Weak negative correlation	−0.460 (0.114)	Non-significant—Weak negative correlation
9.	TFC ^c^	FRAP ^e^	0.240 (0.430)	Non-significant—Very weak positive correlation	0.348 (0.245)	Non-significant—Weak positive correlation
10.	IC_50_^d^	FRAP ^e^	−0.777 (0.002)	Significant—Strong negative correlation	−0.806 (0.001)	Significant—Strong negative correlation

^a^ RA (µg/mL), ^b^ TPC (mg GAE/mL), GAE, gallic acid equivalent; ^c^ TFC (mg QE/mL), QE, quercetin equivalent; ^d^ IC_50_ (mg/mL), and ^e^ FRAP (mg TE/mL), TE, Trolox equivalent. **Abbreviations:** RA, rosmarinic acid; TPC, total phenolic content; TFC, total flavonoid content; IC_50_, half-maximal inhibitory concentration; FRAP, ferric reducing antioxidant power.

**Table 10:** Geographical and varietal statistical comparison of phytochemical and antioxidant parameters.

Entry	Factor	Parameter	Test Used	Effect Size (η^2^/ε^2^)	*p*-Value	Group Difference
1.	Geo-climatic Zone	RA ^a^	Kruskal–Wallis test	0.87	0.033	Significant
2.	Geo-climatic Zone	TPC ^b^	One-way ANOVA	0.68	0.039	Significant
3.	Geo-climatic Zone	TFC ^c^	One-way ANOVA	0.61	0.078	Non-significant
4.	Geo-climatic Zone	IC_50_^d^	One-way ANOVA	0.77	0.012	Significant
5.	Geo-climatic Zone	FRAP ^e^	One-way ANOVA	0.74	0.017	Significant
6.	Variety	RA ^a^	Kruskal–Wallis test	0.00	1.000	Non-significant
7.	Variety	TPC ^b^	One-way ANOVA	0.03	0.591	Non-significant
8.	Variety	TFC ^c^	One-way ANOVA	0.02	0.644	Non-significant
9.	Variety	IC_50_^d^	One-way ANOVA	0.00	0.854	Non-significant
10.	Variety	FRAP ^e^	One-way ANOVA	0.00	0.917	Non-significant

^a^ RA (µg/mL), ^b^ TPC (mg GAE/mL), ^c^ TFC (mg QE/mL), ^d^ IC_50_ (mg/mL), and ^e^ FRAP (mg TE/mL). GAE, gallic acid equivalent; QE, quercetin equivalent; TE, Trolox equivalent; ANOVA, analysis of variance; η^2^, effect size for ANOVA; ε^2^, effect size for Kruskal–Wallis test. **Abbreviations:** RA, rosmarinic acid; TPC, total phenolic content; TFC, total flavonoid content; IC_50_, half-maximal inhibitory concentration; FRAP, ferric reducing antioxidant power.

**Table 11:** Collection details of field and market samples from different geo-climatic regions of India.

Entry	Sample	Field No	Variety	Geo-Climatic Zone	Date of Collection	GPS ^1^
1.	Field Sample-1	10612	Non-Krishna (NK)	Western Ghats (W)	10.04.2024	Lat. 12.255520°, Long. 78.064314°
2.	Field Sample-2	10835	Krishna	Gangetic Plain (G)	04.02.2025	Lat. 22.971838°, Long. 88.436743°
3.	Field Sample-3	10844	Non-Krishna (NK)	Gangetic Plain (G)	04.02.2025	Lat. 22.971838°, Long. 88.436743°
4.	Field Sample-4	10876	Krishna	Eastern Ghats (E)	13.02.2025	Lat. 11.415507°, Long. 77.229156°
5.	Field Sample-5	10909	Non-Krishna (NK)	Deccan Region (D)	22.02.2025	Lat. 17.123863°, Long. 79.365835°
6.	Field Sample-6	11009	Non-Krishna (NK)	Western Ghats (W)	28.06.2025	Lat. 8.311533°, Long. 77.442415°
7.	Field Sample-7	11018	Krishna	Western Ghats (W)	29.06.2025	Lat. 8.364615°, Long. 77.296771°
8.	Field Sample-8	11114	Krishna	Indus Plain/Semi-Arid (I)	16.10.2025	Lat. 22.286668°, Long. 73.183116°
9.	Field Sample-9	11118	Non-Krishna (NK)	Indus Plain/Semi-Arid (I)	17.10.2025	Lat. 22.579330°, Long. 72.933903°
10.	Market Sample-1	Not Applicable	Non-Krishna (NK)	Gangetic Plain (G)	05.02.2025 ^2^	Not Applicable ^3^
11.	Market Sample-2	Not Applicable	Non-Krishna (NK)	Deccan Region (D)	19.02.2025 ^2^	Not Applicable ^3^
12.	Market Sample-3	Not Applicable	Non-Krishna (NK)	Eastern Ghats (E)	30.01.2025 ^2^	Not Applicable ^3^
13.	Market Sample-4	Not Applicable	Non-Krishna (NK)	Indus Plain/Semi-Arid (I)	16.10.2025 ^2^	Not Applicable ^3^

^1^ GPS, Global Positioning System. ^2^ It is the date of purchase/collection from the vendor, not the date of field collection. ^3^ The exact cultivation/growing location of the plant materials was not available.

## Data Availability

The original contributions presented in this study are included in the article and its Supplementary Material. Further inquiries can be directed to the corresponding author.

## Supplementary Materials

The following supporting information can be downloaded at: https://www.mdpi.com/article/10.3390/molecules31213909/s1, Figure S1. Molecular docking interaction surface visualization of rosmarinic acid (RA), ursolic acid (UA), and reference ligand complexes with target proteins 5IKT, 3LN1, and 4KFB; Figure S2. Molecular docking interaction surface visualization of rosmarinic acid (RA), ursolic acid (UA), and reference ligand complexes with target proteins 1M17, 1O86, 2V5Z, and 3NVY; Figure S3. Calibration curve of rosmarinic acid (RA) obtained by HPTLC densitometry at 325 nm; Figure S4. ESI(−)-MS spectrum of purified rosmarinic acid (RA); Figure S5. ^13^C- NMR Spectrum of purified rosmarinic acid (RA) in d_4_-Methanol^;^ Figure S6. ^1^H- NMR Spectrum of purified rosmarinic acid (RA) in d_4_-Methanol; Figure S7. Standard calibration curve used for total phenolic content (TPC) determination; Figure S8. Standard calibration curve used for total flavonoid content (TFC) determination; Figure S9. Standard calibration curve used for Ferric Reducing Antioxidant Power (FRAP) determination; Figure S10. Plant habit and field collection of *Ocimum tenuiflorum* L. across different natural habitats: Habit and field collection in the Western Ghats, *Ocimum tenuiflorum* var. *krishna* (Tulsi) habit in the Gangetic Plain, *Ocimum tenuiflorum* var. *krishna* (Tulsi) habit in the Indus Plain, *Ocimum tenuiflorum* habit in the Eastern Ghats, *Ocimum tenuiflorum* L. habit in the Indus Plain, and *Ocimum tenuiflorum* L. habit in the Deccan Plateau; Figure S11a. Herbarium specimen of Field Book No.: 63; Field No.: 10612; Accession No.: 11750; Date of Collection: 10.04.2024; Common Name / Local Name: Tulasi; Botanical Name: *Ocimum tenuiflorum* L.; Family: Lamiaceae; Habit: Herb; Parts Collected: Aerial Part; GPS: Lat. 12.255520°, Long. 78.064314°; District / State: Dharmapuri, Tamil Nadu; Locality: Bhellaralli, on the way to Karimangalam; Collector’s Name:. M. Murali; Authenticated by: J. Shashikanth; Figure S11b. Herbarium specimen of Field Book No.: 64; Field No.: 10835; Accession No.: 12059; Date of Collection: 04.02.2025; Common Name / Local Name: Krishna tulsi; Botanical Name: *Ocimum tenuiflorum* L. var. *krishna* tulsi; Family: Lamiaceae; Habit: Herb; Parts Collected: Aerial Part; GPS: Lat. 22.971838°, Long. 88.436743°; District / State: Nadia, West Bengal; Locality: Kalyani Village; Collector’s Name: S. Mugendhiran, Shri. M. Murali and V. Manimekala; Authenticated by: J. Shashikanth; Figure S11c. Herbarium specimen of Field Book No.: 64; Field No.: 10844; Accession No.: 12069; Date of Collection: 04.02.2025; Common Name / Local Name: Tulsi; Botanical Name: *Ocimum tenuiflorum* L.; Family: Lamiaceae; Habit: Herb; Parts Collected: Aerial part; GPS: Lat. 22.971838°, Long. 88.436743°; District / State: Nadia, West Bengal; Locality: Kalyani village; Collector’s Name: Shri. S. Mugendhiran, M. Murali and V. Manimekala; Authenticated by: J. Shashikanth; Figure S11d. Herbarium specimen of Field Book No.: 64; Field No.: 10876; Accession No.: 12112; Date of Collection: 13.02.2025; Common Name / Local Name: Krishna tulsi; Botanical Name: *Ocimum tenuiflorum* var. Krishna thulasi; Family: Lamiaceae; Habit: Herb; Parts Collected: Aerial part; GPS: Lat. 11.415507°, Long. 77.229156°; District / State: Erode, Tamil Nadu; Locality: Ukkaram Village on the way to Gobichettipalayam; Collector’s Name: J. Shashikanth; Authenticated by: J. Shashikanth; Figure S11e. Herbarium specimen of Field Book No.: 64; Field No.: 10909; Accession No.: 12160; Date of Collection: 22.02.2025; Common Name / Local Name: Tulasi; Botanical Name: *Ocimum tenuiflorum* L.; Family: Lamiaceae; Habit: Herb; Parts Collected: Aerial Part; GPS: Lat. 17.123863°, Long. 79.365835°; District / State: Nalgonda, Telangana; Locality: Chinna Suraram Village; Collector’s Name: J. Shashikanth; Authenticated by: J. Shashikanth; Figure S11f. Herbarium specimen of Field Book No.: 64; Field No.: 11009; Accession No.: 12283; Date of Collection: 28.06.2025; Common Name / Local Name: Tulsi; Botanical Name: *Ocimum tenuiflorum* L.; Family: Lamiaceae; Habit: Herb; Parts Collected: Aerial part; GPS: Lat. 8.311533°, Long. 77.442415°; District / State: Kanniyakumari, Tamil Nadu; Locality: Arumallur Village; Collector’s Name: J. Shashikanth; Authenticated by: J. Shashikanth; Figure S11g. Herbarium specimen of Field Book No.: 64; Field No.: 11018; Accession No.: 12294; Date of Collection: 29.06.2025; Common Name / Local Name: Krishna tulsi; Botanical Name: *Ocimum tenuiflorum* var. Krishna thulasi; Family: Lamiaceae; Habit: Herb; Parts Collected: Aerial part; GPS: Lat. 8.364615°, Long. 77.296771°; District / State: Kanyakumari, Tamil Nadu; Locality: Kulasekaram; Collector’s Name: J. Shashikanth; Authenticated by: J. Shashikanth; Figure S11h. Herbarium specimen of Field Book No.: 65; Field No.: 11114; Accession No.: 12389; Date of Collection: 16.10.2025; Common Name / Local Name: Krishna tulsi; Botanical Name: *Ocimum tenuiflorum* var. Krishna thulasi; Family: Lamiaceae; Habit: Herb; Parts Collected: Aerial part; GPS: Lat. 22.286668°, Long. 73.183116°; District / State: Vadodara (Baroda), Gujarat; Locality: Arboretum cum Medicinal Plant Garden, M.S. University, Vadodara (Baroda); Collector’s Name: J. Shashikanth; Authenticated by: J. Shashikanth; Figure S11i. Herbarium specimen of Voucher ID: 11118; Field Book No.: 65; Field No.: 11118; Accession No.: 12395; Date of Collection: 17.10.2025; Common Name / Local Name: Tulasi; Botanical Name: *Ocimum tenuiflorum* L.; Family: Lamiaceae; Habit: Herb; Parts Collected: Aerial Part; GPS: Lat. 22.579330°, Long. 72.933903°; District / State: Anand, Gujarat; Locality: Lambhvel Village, Anand; Collector’s Name: J. Shashikanth; Authenticated by: J. Shashikanth.

## Abbreviations

The following abbreviations are used in this manuscript:

**Abbreviation**	**Full Form**
4PL	Four-Parameter Logistic Regression
ASR	Anisaldehyde Sulphuric Acid Reagent
DCM	Dichloromethane
DPPH	2,2-Diphenyl-1-picrylhydrazyl
FRAP	Ferric Reducing Antioxidant Power
GAE	Gallic Acid Equivalent
GC–MS/MS	Gas Chromatography Tandem Mass Spectrometry
HCA	Hierarchical Cluster Analysis
HPTLC	High-Performance Thin-Layer Chromatography
HRMS	High-Resolution Mass Spectrometry
ICH	International Council for Harmonisation
IC_50_	Half-Maximal Inhibitory Concentration
LC–MS/MS	Liquid Chromatography Tandem Mass Spectrometry
NK	Non-Krishna
NP/PEG	Natural Product/Polyethylene Glycol Derivatization Reagent
OT	*Ocimum tenuiflorum*
PCA	Principal Component Analysis
PDB	Protein Data Bank
PREs	Pharmacopeia-Relevant Extracts
QE	Quercetin Equivalent
R^2^	Coefficient of Determination
RA	Rosmarinic Acid
ROS	Reactive Oxygen Species
RSD	Relative Standard Deviation
R_f_	Retention Factor
RMSD	Root Mean Square Deviation
TE	Trolox Equivalent
TFC	Total Flavonoid Content
TPC	Total Phenolic Content
UA	Ursolic Acid
UV–Vis	Ultraviolet–Visible Spectroscopy
