# Supplementary material for: Integrated Phytochemical, Antioxidant, and Chemometric Evaluation of Clinically Relevant Ocimum tenuiflorum Extracts from Diverse Geo-Climatic Regions
Source: Molecules. 2026 Oct 29;31(21):3909. doi: 10.3390/molecules31213909 (PMC13765221; doi:10.3390/molecules31213909)
Supplement: Supplementary file 1 [file molecules-31-03909-s001.zip › molecules-4562512-supplementary.pdf]

Integrated Phytochemical, Antioxidant, and Chemometric Evaluation of Clinically Relevant *Ocimum tenuiflorum* Extracts from Diverse Geo-Climatic Regions

Supplementary Materials

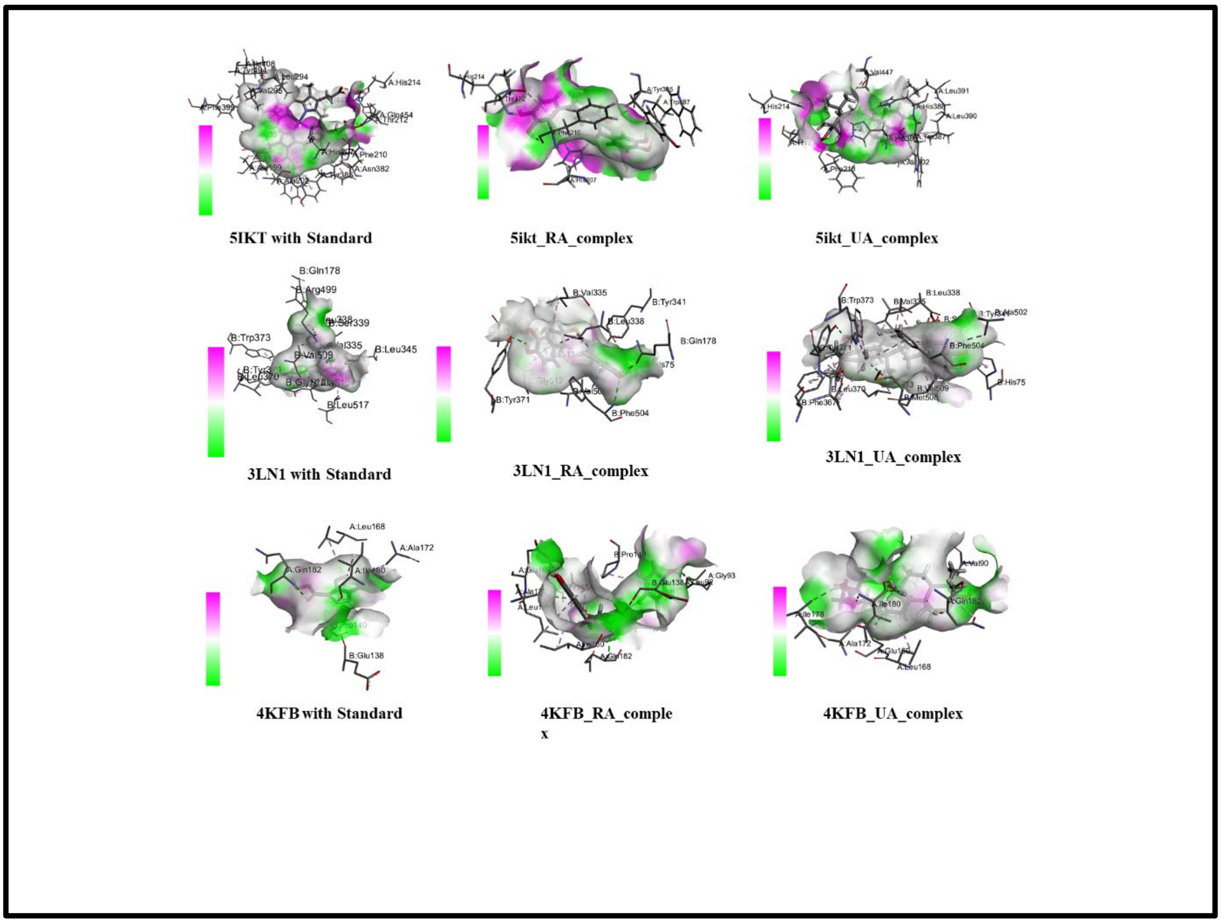

Figure S1. Molecular docking interaction surface visualization of rosmarinic acid (RA), ursolic acid (UA), and reference ligand complexes with target proteins 5IKT, 3LN1, and 4KFB.

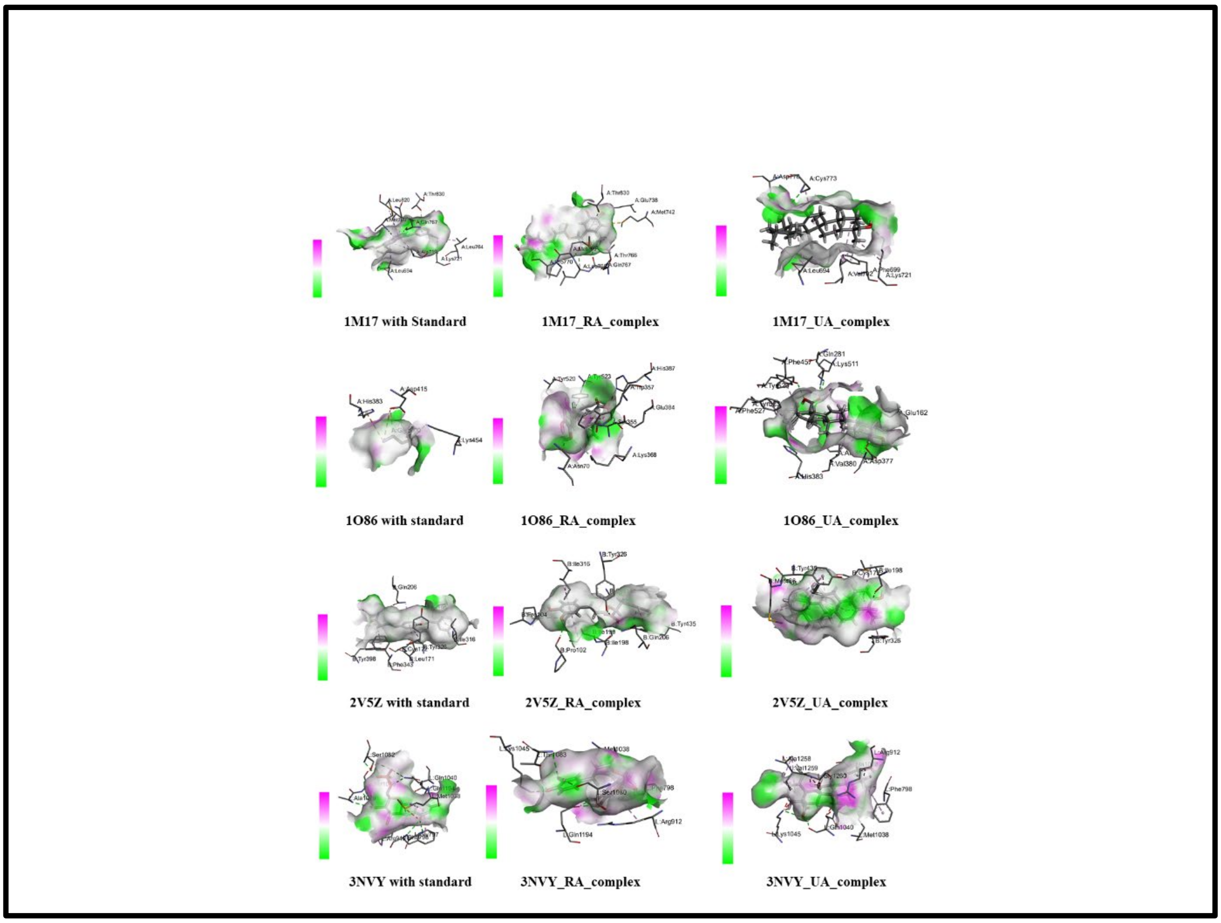

Figure S2. Molecular docking interaction surface visualization of rosmarinic acid (RA), ursolic acid (UA), and reference ligand complexes with target proteins 1M17, 1O86, 2V5Z, and 3NVY.

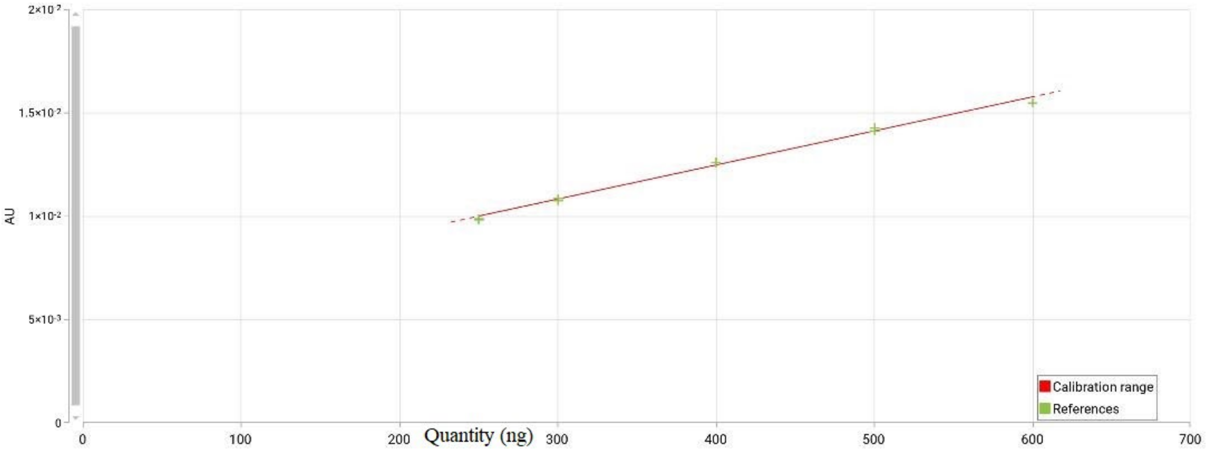

Figure S3. Calibration curve of rosmarinic acid (RA) obtained by HPTLC densitometry at 325 nm.

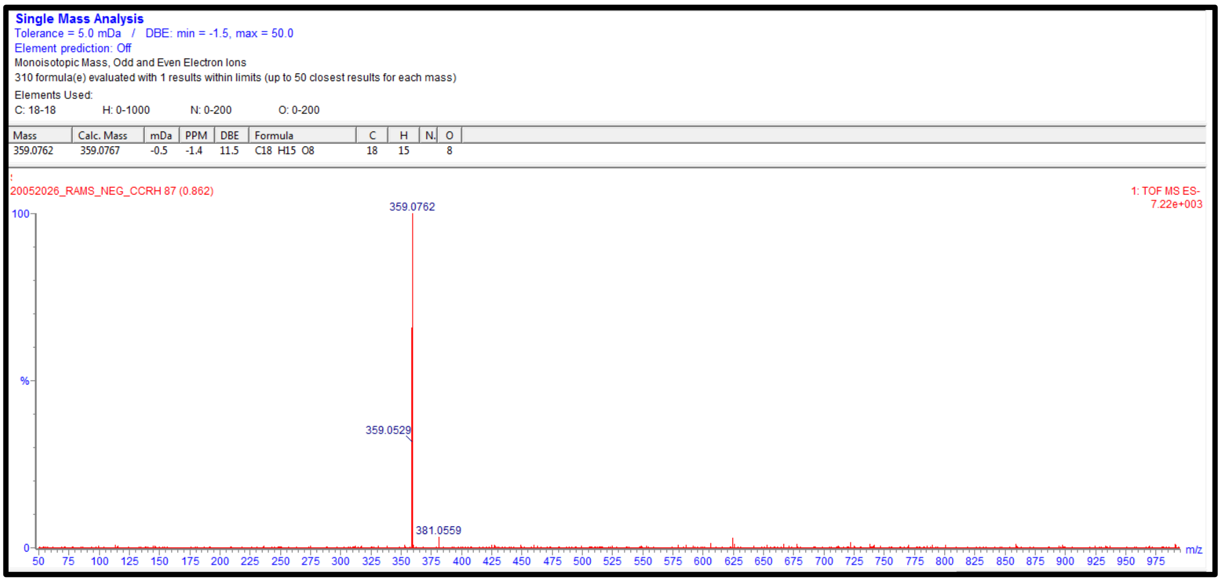

Figure S4. ESI(−)-MS spectrum of purified rosmarinic acid (RA).

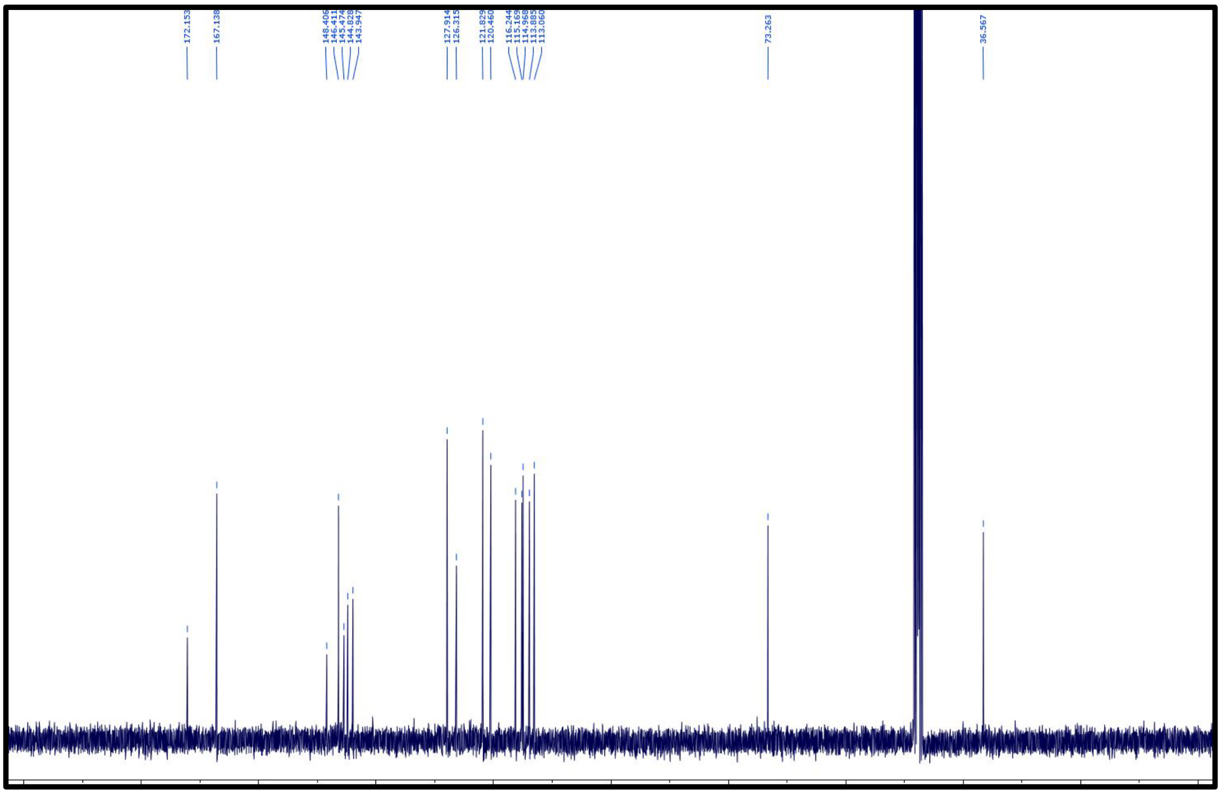

Figure S5. ^{13}C- NMR Spectrum of purified rosmarinic acid (RA) in d_4-Methanol.

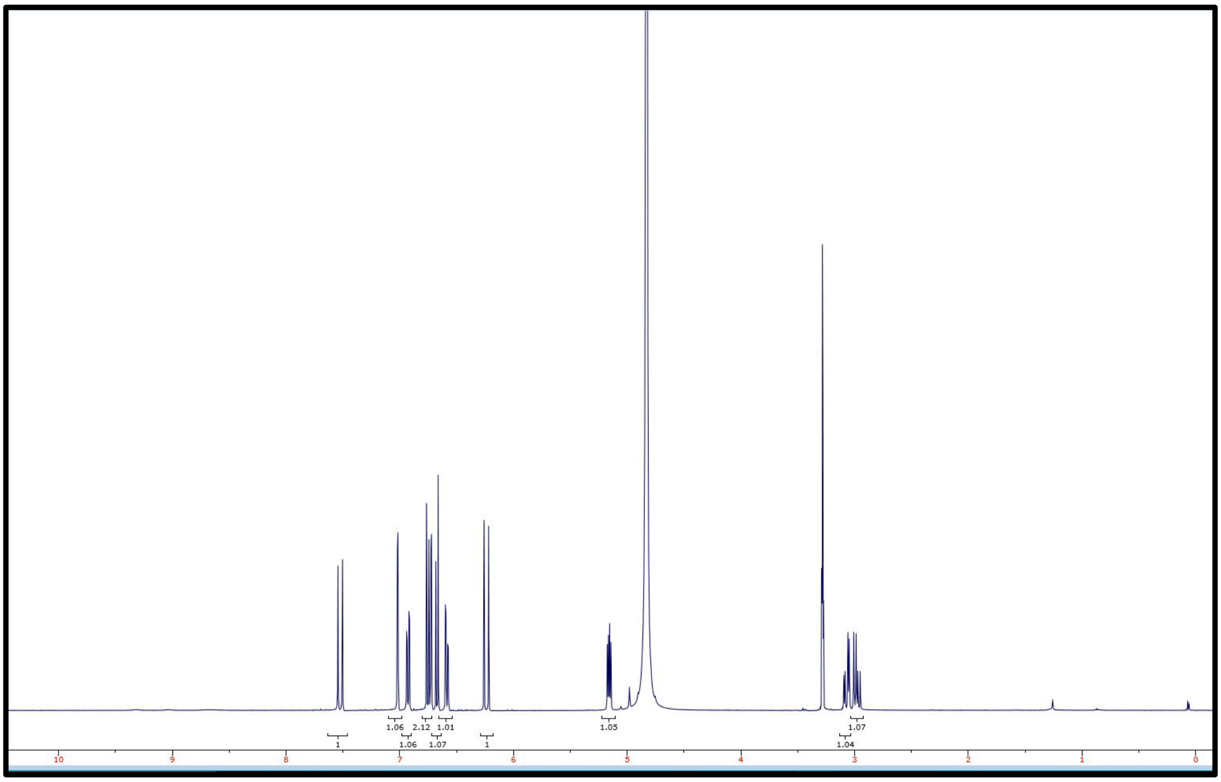

Figure S6. ^{1}H- NMR Spectrum of purified rosmarinic acid (RA) in d_4-Methanol.

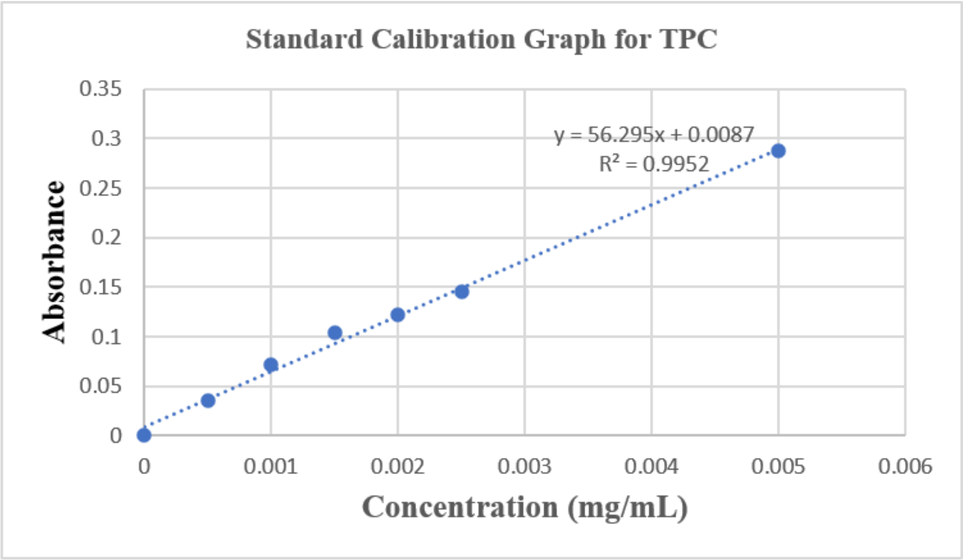

Figure S7. Standard calibration curve used for total phenolic content (TPC) determination.

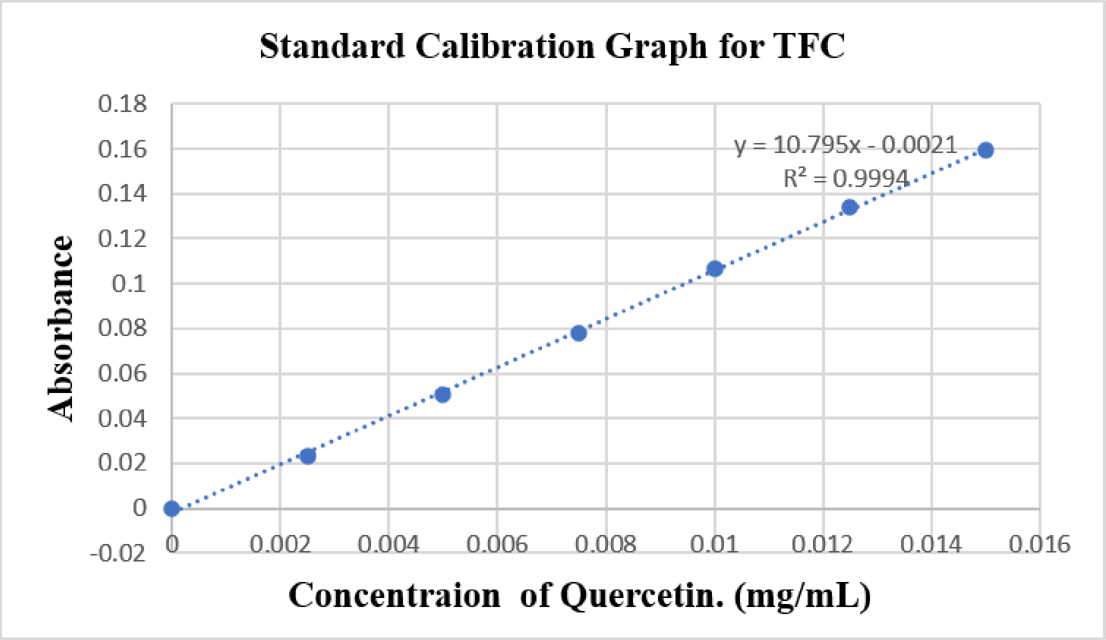

Figure S8. Standard calibration curve used for total flavonoid content (TFC) determination.

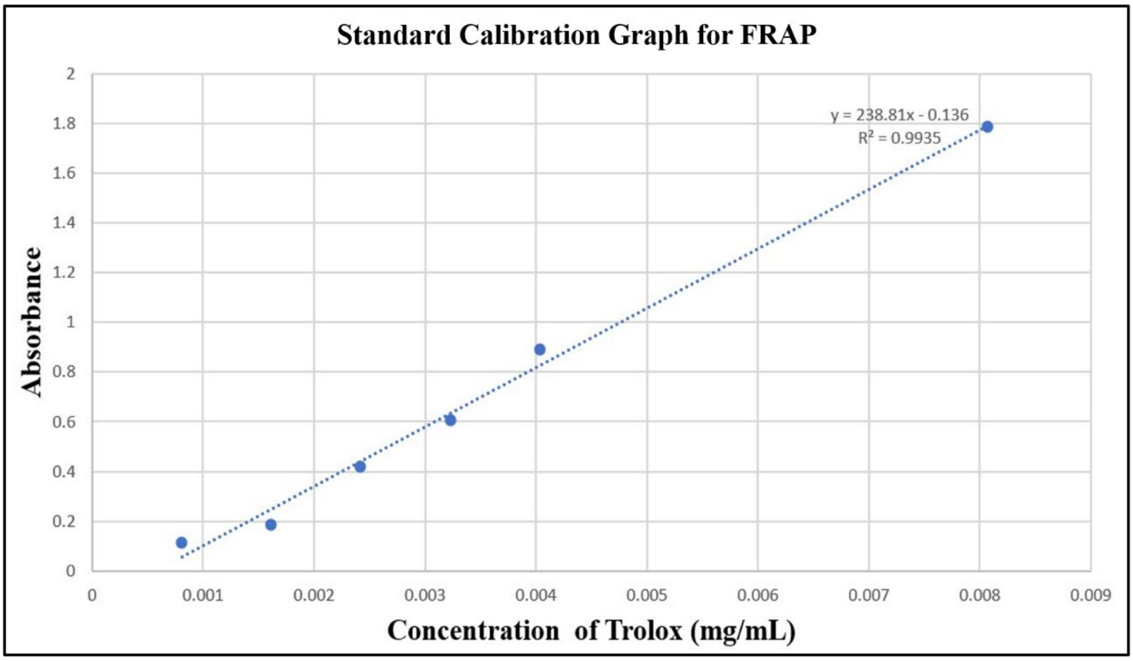

Figure S9. Standard calibration curve used for Ferric Reducing Antioxidant Power (FRAP) determination.

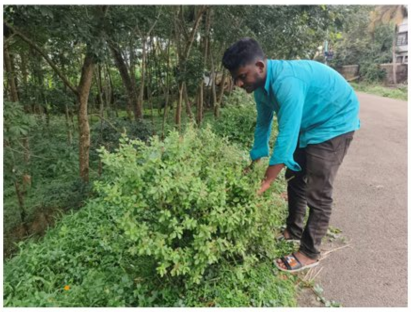

***Ocimum tenuiflorum* L. – Habit and field collection – Western Ghats**

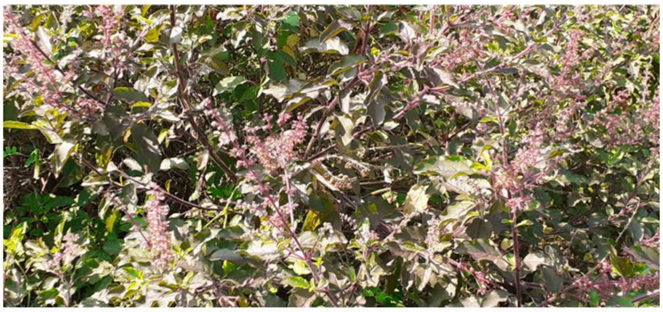

***Ocimum tenuiflorum* var. *krishna* (Tulsi) – Habit – Gangetic Plain**

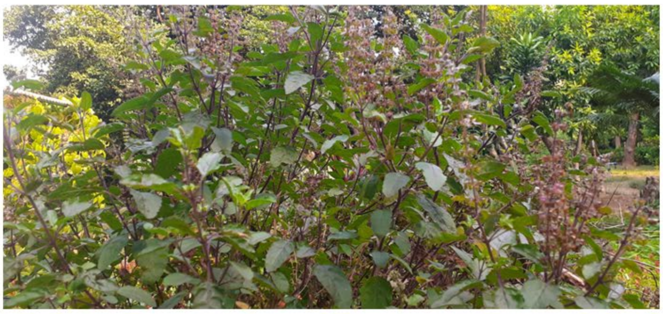

***Ocimum tenuiflorum* L. var. *krishna* (Tulsi) – Habit – Indus Plain**

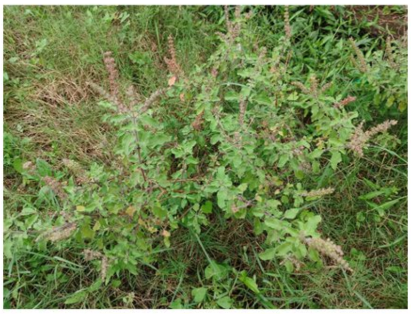

***Ocimum tenuiflorum* L. – Habit – Eastern Ghats**

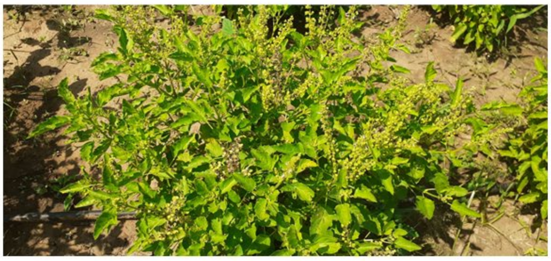

***Ocimum tenuiflorum* L. – Habit – Indus Plain**

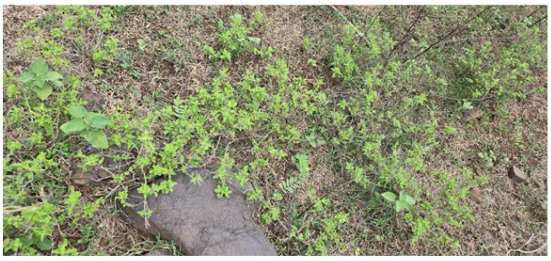

***Ocimum tenuiflorum* L. - Habit –Deccan Region**

Figure S10. Plant habit and field collection of *Ocimum tenuiflorum* L. across different natural habitats: Habit and field collection in the Western Ghats, *Ocimum tenuiflorum* var. *krishna* (Tulsi) habit in the Gangetic Plain, *Ocimum tenuiflorum* var. *krishna* (Tulsi) habit in the Indus Plain, *Ocimum tenuiflorum* habit in the Eastern Ghats, *Ocimum tenuiflorum* L. habit in the Indus Plain, and *Ocimum tenuiflorum* L. habit in the Deccan Plateau.

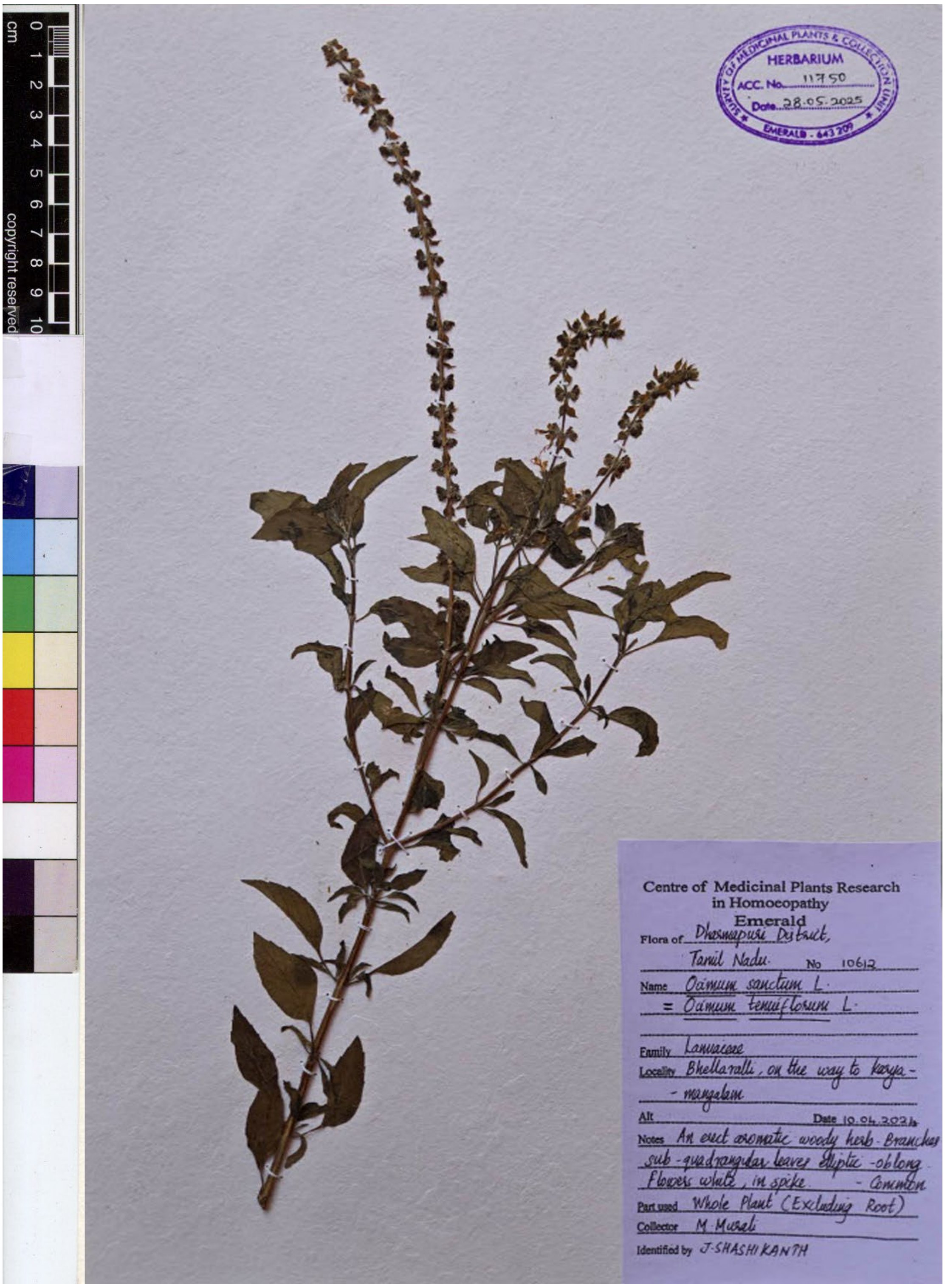

Figure S11a. Herbarium specimen of Field Book No.: 63; **Field No.:** 10612; **Accession No.:** 11750; **Date of Collection:** 10.04.2024; **Common Name / Local Name:** Tulasi; **Botanical Name:** *Ocimum tenuiflorum* L.; **Family:** Lamiaceae; **Habit:** Herb; **Parts Collected:** Aerial Part; **GPS:** Lat. 12.255520°, Long. 78.064314°; **District / State:** Dharmapuri, Tamil Nadu; **Locality:** Bhellaralli, on the way to Karimangalam; **Collector's Name:**. M. Murali; **Authenticated by:** J. Shashikanth

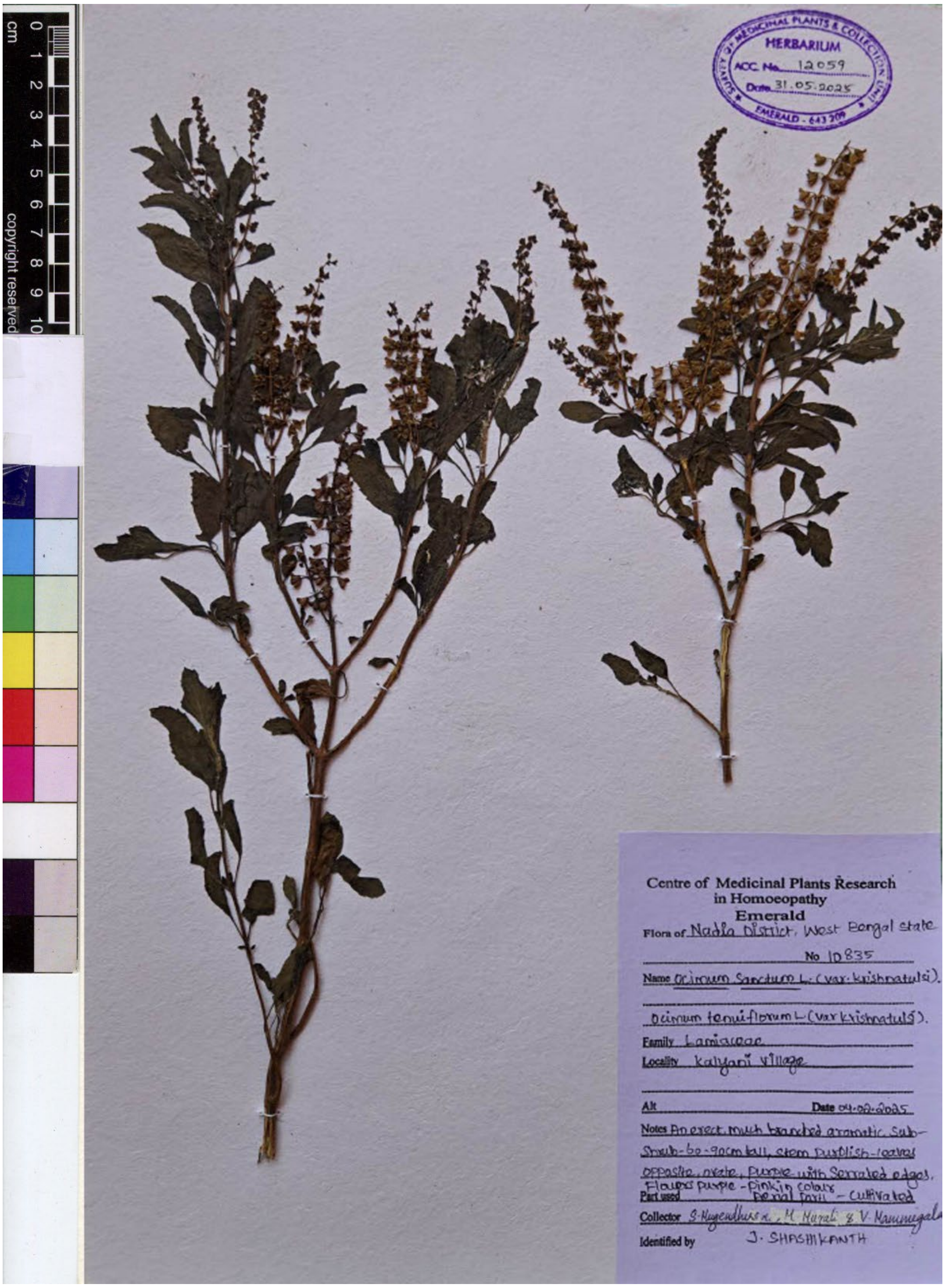

Figure S11b. Herbarium specimen of Field Book No.: 64; **Field No.:** 10835; **Accession No.:** 12059; **Date of Collection:** 04.02.2025; **Common Name / Local Name:** Krishna tulsi; **Botanical Name:** *Ocimum tenuiflorum* L. var. *krishna tulsi*; **Family:** Lamiaceae; **Habit:** Herb; **Parts Collected:** Aerial Part; **GPS:** Lat. 22.971838°, Long. 88.436743°; **District / State:** Nadia, West Bengal; **Locality:** Kalyani Village; **Collector's Name:** S. Mugendhiran, Shri. M. Murali and V. Manimekala; **Authenticated by:** J. Shashikanth

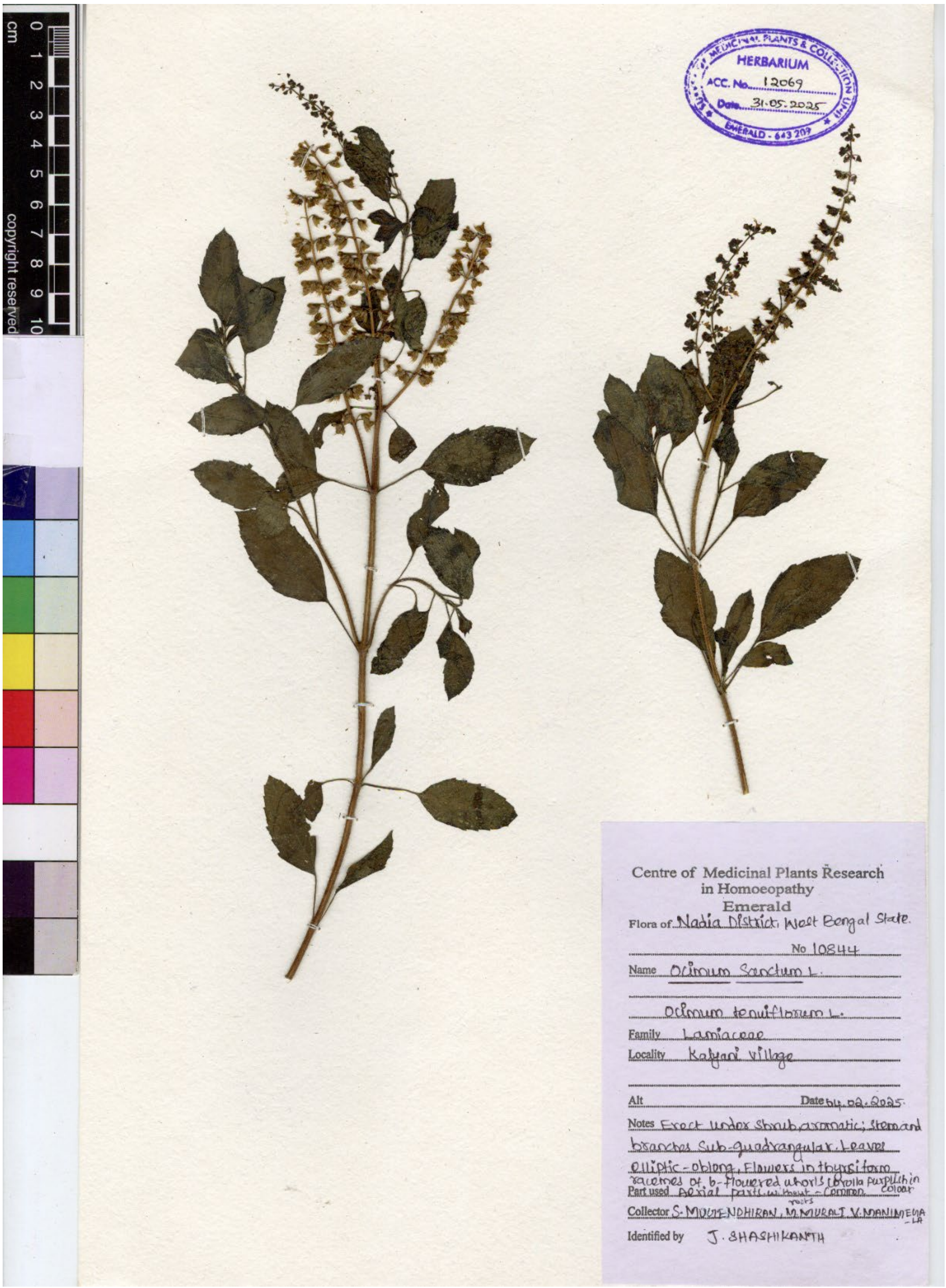

Figure S11c. Herbarium specimen of Field Book No.: 64; **Field No.:** 10844; **Accession No.:** 12069; **Date of Collection:** 04.02.2025; **Common Name / Local Name:** Tulsi; **Botanical Name:** *Ocimum tenuiflorum* L.; **Family:** Lamiaceae; **Habit:** Herb; **Parts Collected:** Aerial part; **GPS:** Lat. 22.971838°, Long. 88.436743°; **District / State:** Nadia, West Bengal; **Locality:** Kalyani village; **Collector's Name:** Shri. S. Mugendhiran, M. Murali and V. Manimekala; **Authenticated by:** J. Shashikanth

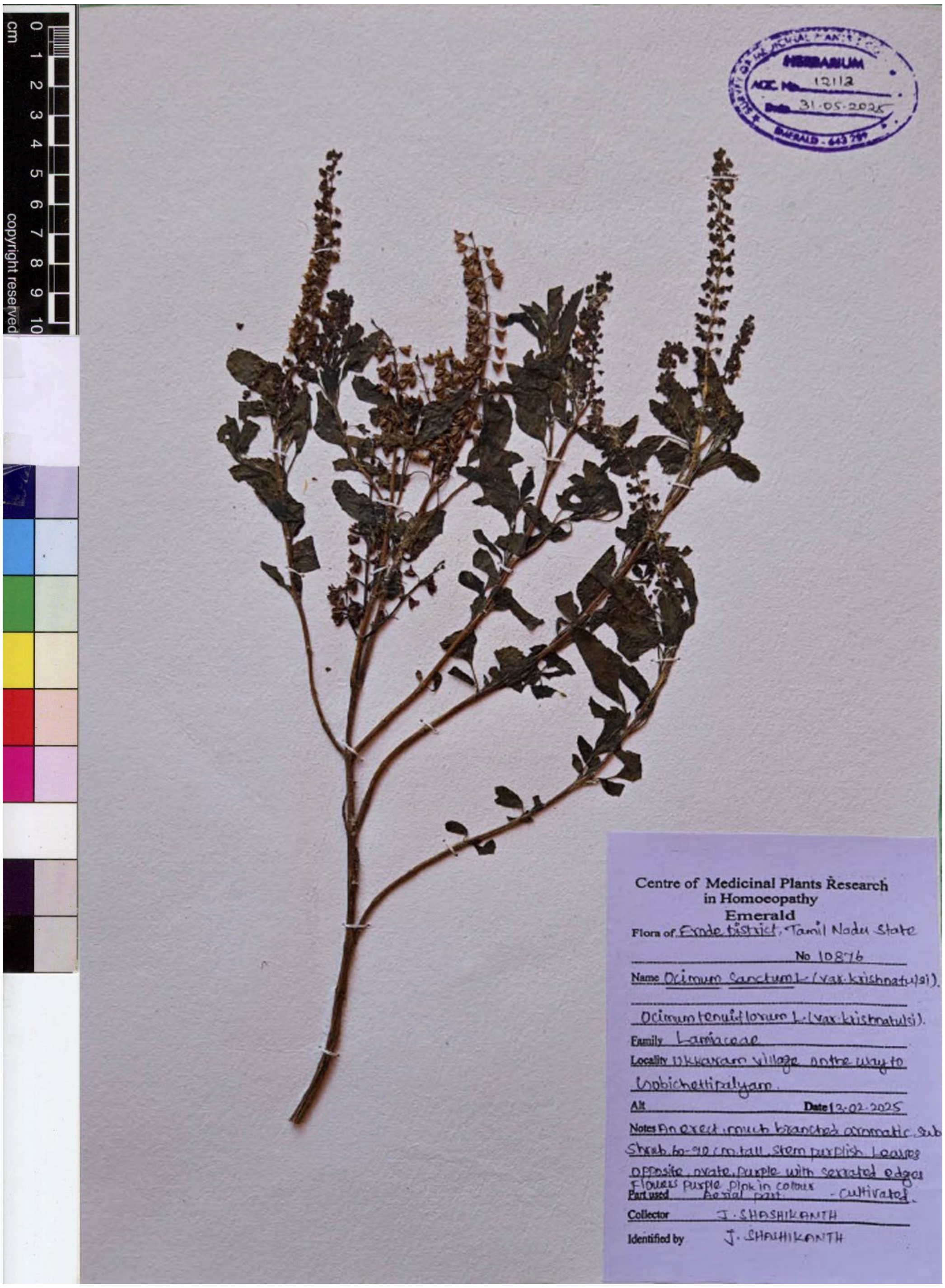

Figure S11d. Herbarium specimen of Field Book No.: 64; **Field No.:** 10876; **Accession No.:** 12112; **Date of Collection:** 13.02.2025; **Common Name / Local Name:** Krishna tulsi; **Botanical Name:** *Ocimum tenuiflorum* var. *Krishna thulasi*; **Family:** Lamiaceae; **Habit:** Herb; **Parts Collected:** Aerial part; **GPS:** Lat. 11.415507°, Long. 77.229156°; **District / State:** Erode, Tamil Nadu; **Locality:** Ukkaram Village on the way to Gobichettipalayam; **Collector's Name:** J. Shashikanth; **Authenticated by:** J. Shashikanth

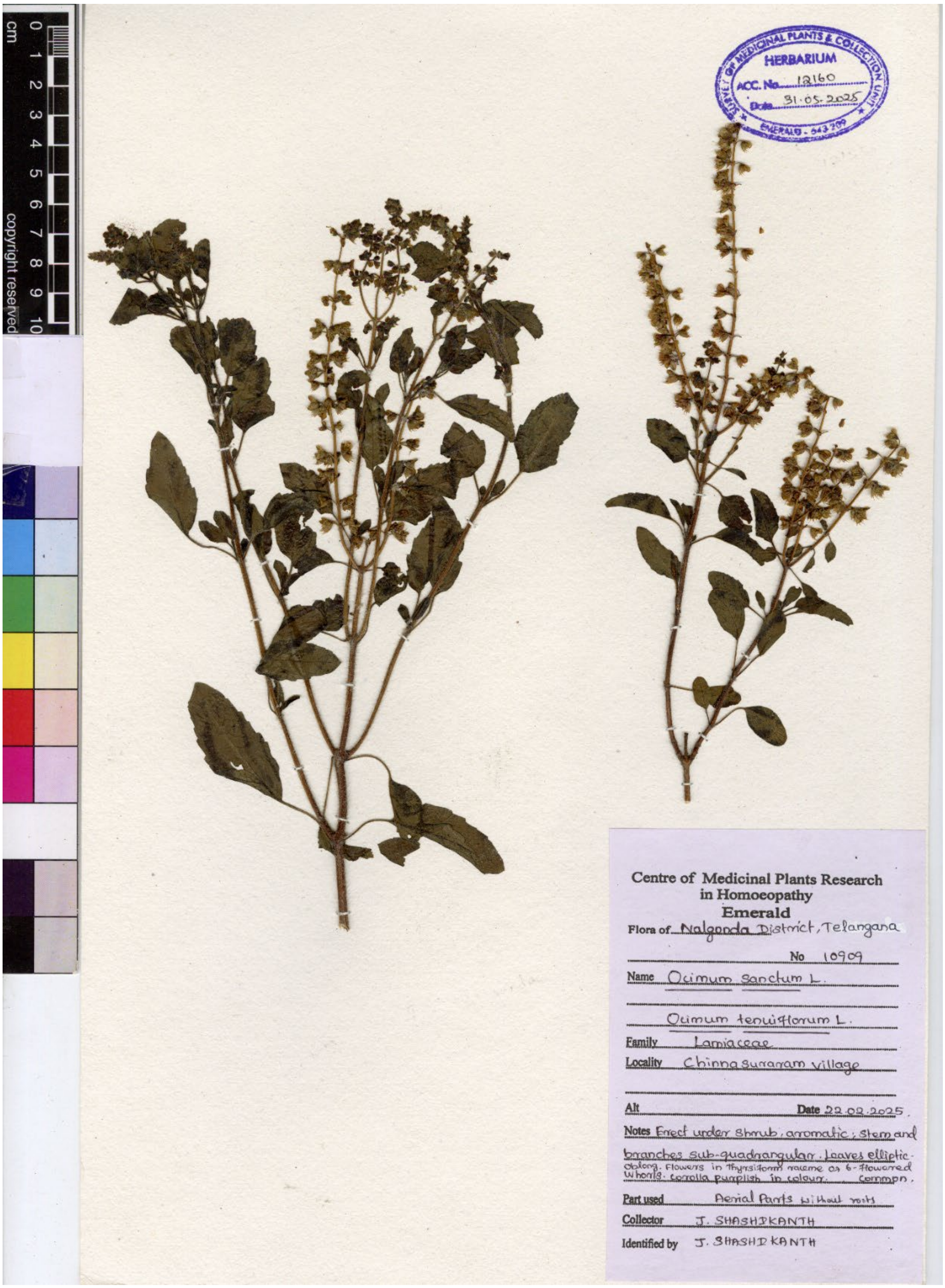

Figure S11e. Herbarium specimen of Field Book No.: 64; **Field No.:** 10909; **Accession No.:** 12160; **Date of Collection:** 22.02.2025; **Common Name / Local Name:** Tulasi; **Botanical Name:** *Ocimum tenuiflorum* L.; **Family:** Lamiaceae; **Habit:** Herb; **Parts Collected:** Aerial Part; **GPS:** Lat. 17.123863°, Long. 79.365835°; **District / State:** Nalgonda, Telangana; **Locality:** Chinna Suraram Village; **Collector's Name:** J. Shashikanth; **Authenticated by:** J. Shashikanth

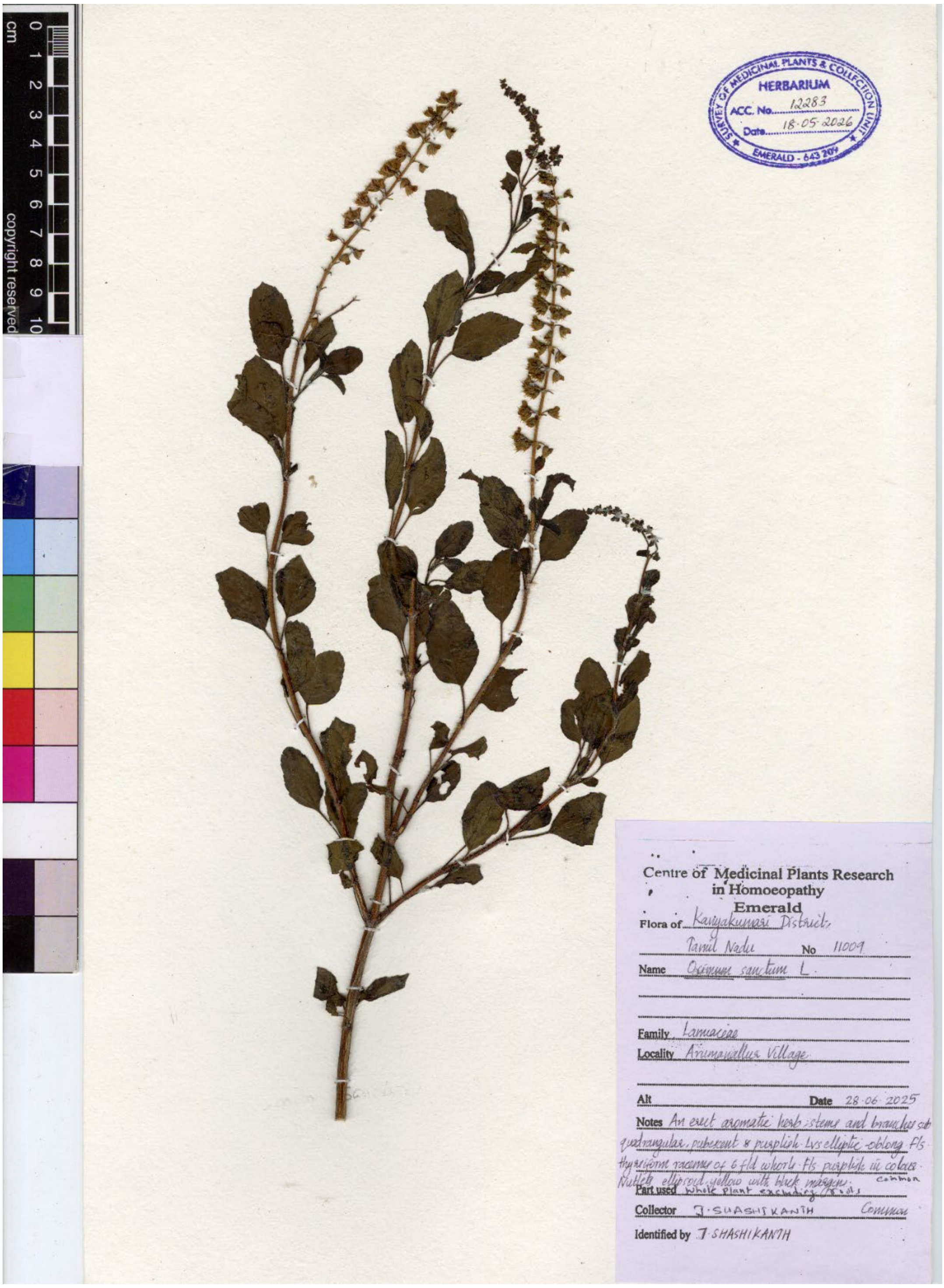

Figure S11f. Herbarium specimen of Field Book No.: 64; **Field No.:** 11009; **Accession No.:** 12283; **Date of Collection:** 28.06.2025; **Common Name / Local Name:** Tulsi; **Botanical Name:** *Ocimum tenuiflorum* L.; **Family:** Lamiaceae; **Habit:** Herb; **Parts Collected:** Aerial part; **GPS:** Lat. 8.311533°, Long. 77.442415°; **District / State:** Kanniyakumari, Tamil Nadu; **Locality:** Arumallur Village; **Collector's Name:** J. Shashikanth; **Authenticated by:** J. Shashikanth

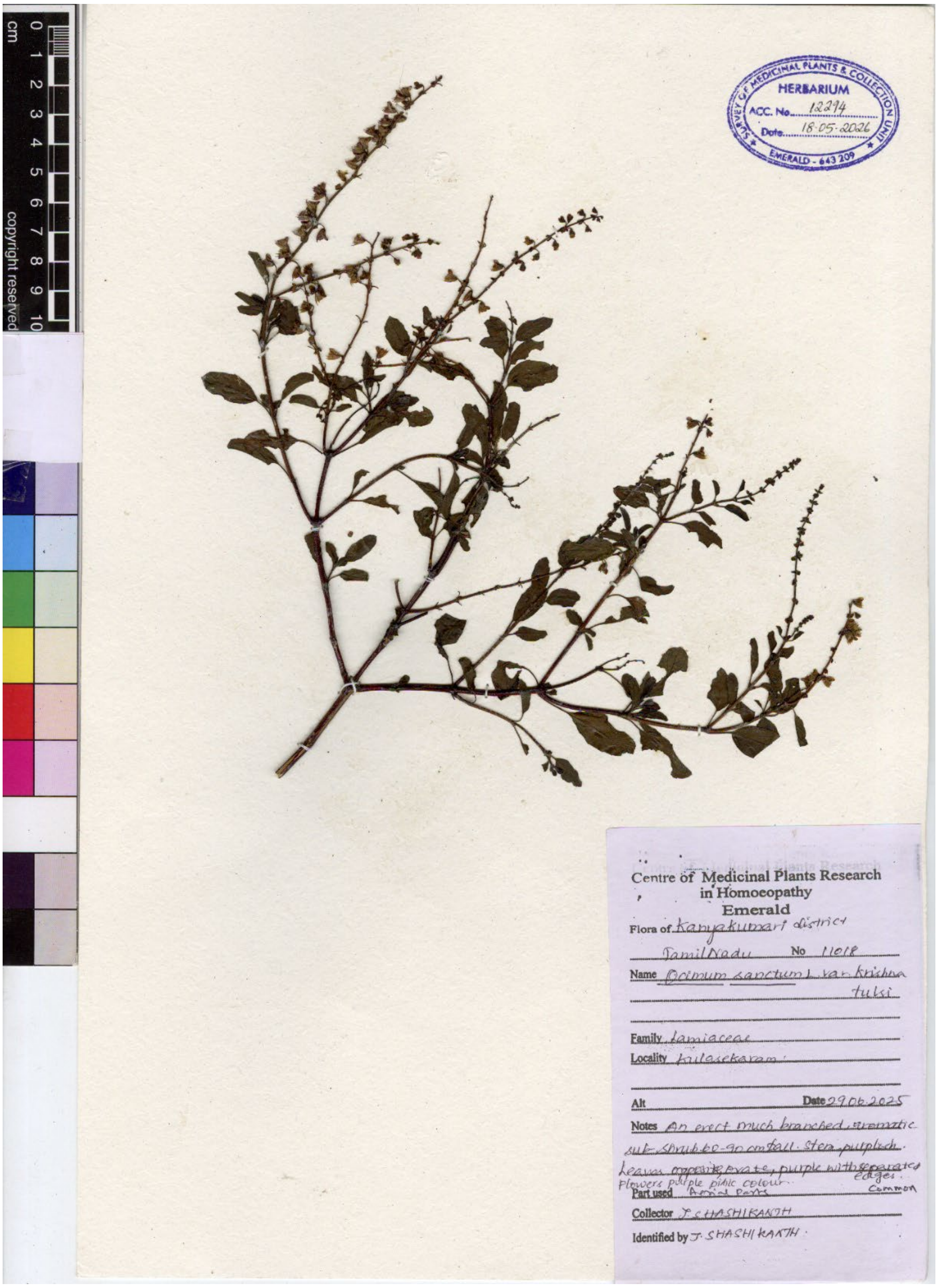

Figure S11g. Herbarium specimen of **Field Book No.:** 64; **Field No.:** 11018; **Accession No.:** 12294; **Date of Collection:** 29.06.2025; **Common Name / Local Name:** Krishna tulsi; **Botanical Name:** *Ocimum tenuiflorum* var. *Krishna thulasi*; **Family:** Lamiaceae; **Habit:** Herb; **Parts Collected:** Aerial part; **GPS:** Lat. 8.364615°, Long. 77.296771°; **District / State:** Kanyakumari, Tamil Nadu; **Locality:** Kulasekaram; **Collector's Name:** J. Shashikanth; **Authenticated by:** J. Shashikanth

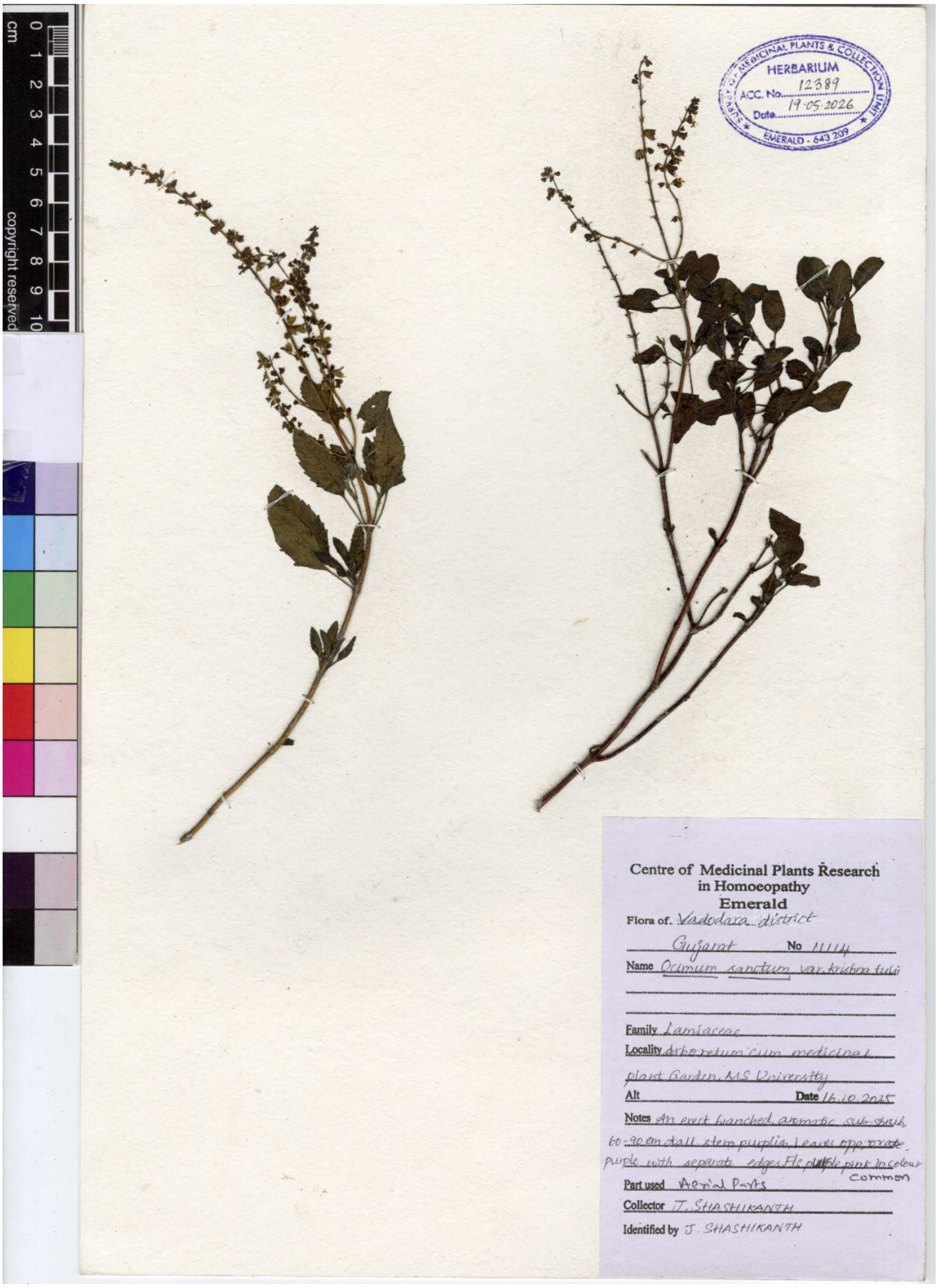

Figure S11h. Herbarium specimen of Field Book No.: 65; **Field No.:** 11114; **Accession No.:** 12389; **Date of Collection:** 16.10.2025; **Common Name / Local Name:** Krishna tulsi; **Botanical Name:** *Ocimum tenuiflorum* var. *Krishna thulasi*; **Family:** Lamiaceae; **Habit:** Herb; **Parts Collected:** Aerial part; **GPS:** Lat. 22.286668°, Long. 73.183116°; **District / State:** Vadodara (Baroda), Gujarat; **Locality:** Arboretum cum Medicinal Plant Garden, M.S. University, Vadodara (Baroda); **Collector's Name:** J. Shashikanth; **Authenticated by:** J. Shashikanth

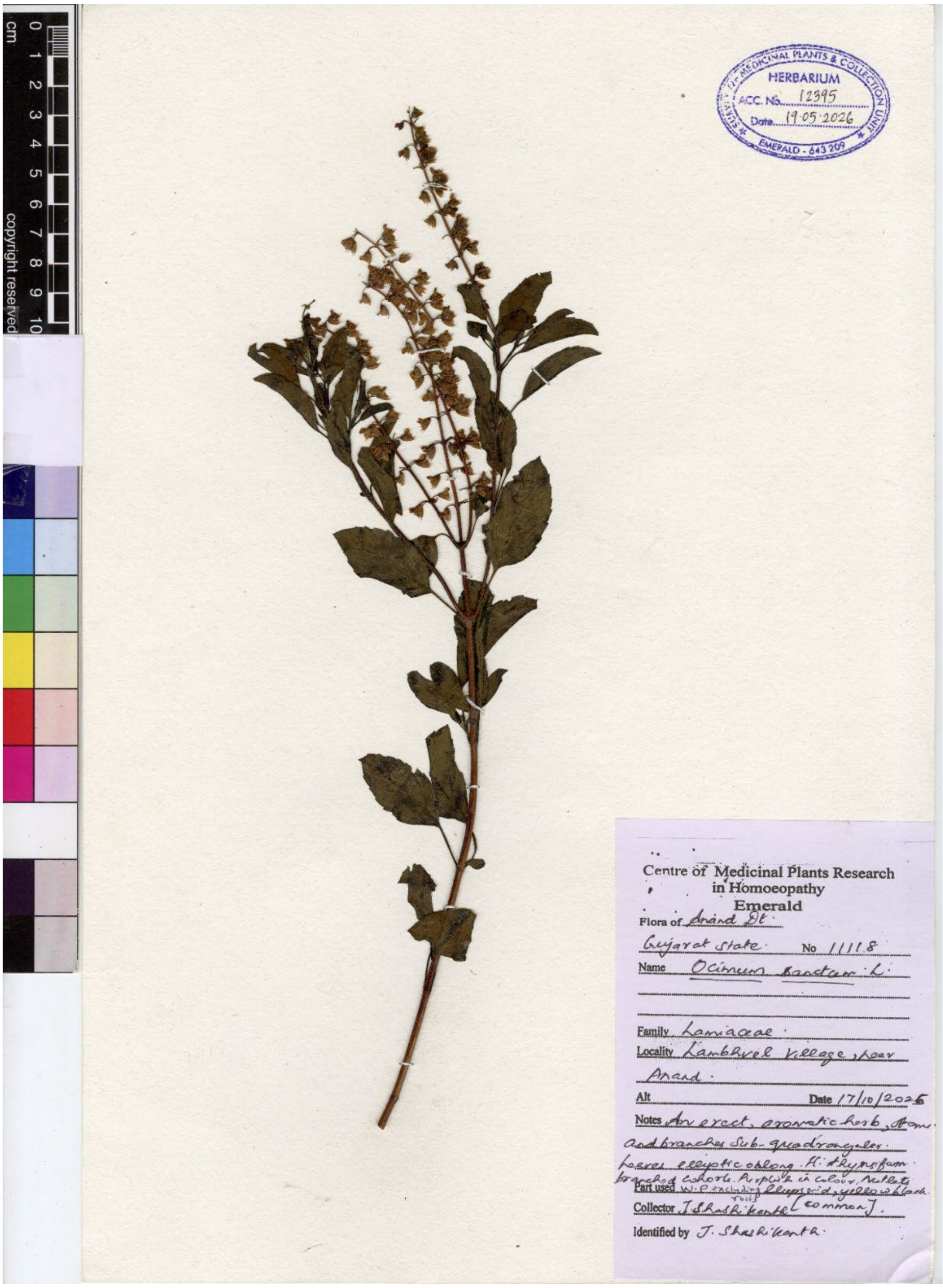

Figure S11i. Herbarium specimen of Voucher ID: 11118; **Field Book No.:** 65; **Field No.:** 11118; **Accession No.:** 12395; **Date of Collection:** 17.10.2025; **Common Name / Local Name:** Tulasi; **Botanical Name:** *Ocimum tenuiflorum* L.; **Family:** Lamiaceae; **Habit:** Herb; **Parts Collected:** Aerial Part; **GPS:** Lat. 22.579330°, Long. 72.933903°; **District / State:** Anand, Gujarat; **Locality:** Lambhvel Village, Anand; **Collector's Name:** J. Shashikanth; **Authenticated by:** J. Shashikanth.
